# Modifiable Risk Factors for Increased Arterial Stiffness in Outpatient Nephrology

**DOI:** 10.1371/journal.pone.0123903

**Published:** 2015-04-16

**Authors:** Usama Elewa, Beatriz Fernandez-Fernandez, Raquel Alegre, Maria D. Sanchez-Niño, Ignacio Mahillo-Fernández, Maria Vanessa Perez-Gomez, Hussein El-Fishawy, Dawlat Belal, Alberto Ortiz

**Affiliations:** 1 IIS-Fundación Jiménez Díaz, School of Medicine, Universidad Autónoma de Madrid, Madrid, Spain; 2 Fundación Renal Iñigo Alvarez de Toledo-IRSIN, Madrid, Spain; 3 REDINREN, Madrid, Spain; 4 Kasr El-Aini University Hospitals, Cairo University, Cairo, Egypt; 5 IDIPaz, Madrid, Spain; Hospital Universitario de La Princesa, SPAIN

## Abstract

Arterial stiffness, as measured by pulse wave velocity (PWV), is an independent predictor of cardiovascular events and mortality. Arterial stiffness increases with age. However, modifiable risk factors such as smoking, BP and salt intake also impact on PWV. The finding of modifiable risk factors may lead to the identification of treatable factors, and, thus, is of interest to practicing nephrologist. We have now studied the prevalence and correlates of arterial stiffness, assessed by PWV, in 191 patients from nephrology outpatient clinics in order to identify modifiable risk factors for arterial stiffness that may in the future guide therapeutic decision-making. PWV was above normal levels for age in 85/191 (44.5%) patients. Multivariate analysis showed that advanced age, systolic BP, diabetes mellitus, serum uric acid and calcium polystyrene sulfonate therapy or calcium-containing medication were independent predictors of PWV. A new parameter, Delta above upper limit of normal PWV (Delta PWV) was defined to decrease the weight of age on PWV values. Delta PWV was calculated as (measured PWV) - (upper limit of the age-adjusted PWV values for the general population). Mean±SD Delta PWV was 0.76±1.60 m/sec. In multivariate analysis, systolic blood pressure, active smoking and calcium polystyrene sulfonate therapy remained independent predictors of higher delta PWV, while age, urinary potassium and beta blocker therapy were independent predictors of lower delta PWV. In conclusion, arterial stiffness was frequent in nephrology outpatients. Systolic blood pressure, smoking, serum uric acid, calcium-containing medications, potassium metabolism and non-use of beta blockers are modifiable factors associated with increased arterial stiffness in Nephrology outpatients.

## Introduction

The elasticity of large arteries moderates systolic pressure peaks and maintains sufficient diastolic pressure to guarantee myocardial perfusion. Under disease conditions natural elasticity may be lost, a condition termed arterial stiffness, leading to high systolic and pulse blood pressure (BP), low diastolic BP, increased left ventricular afterload and lower coronary perfusion. In this regard, arterial stiffness as measured by carotid-femoral pulse wave velocity (PWV) is an independent predictor of cardiovascular (CV) and total mortality and nonfatal CV events in the elderly, the general population and in patients with hypertension, diabetes mellitus (DM), and chronic kidney disease (CKD) [1–4]. Aortic stiffness retains its predictive value for CV events after adjustment to classical risk factors [5, 6] or Framingham risk score [7]. Non-invasive assessment of arterial stiffness has been proposed for individual CV risk evaluation and early detection of vascular damage [8, 9]. Indeed, arterial stiffness has been used as an end-point in interventional studies in CKD patients [10]. PWV has emerged as a gold standard to assess arterial stiffness due to its accuracy, reproducibility, relative easy measurement, low cost and availability of reference values [9].

Arterial stiffness increases with age [2, 9]. In addition, genetic factors, obesity, hypertension, DM, CKD and other factors promoting vascular calcification or altering the protein composition of the vessel wall (decreasing of the elastin/collagen ratio) unfavorably impact on aortic PWV [11–16]. Of these, modifiable risk factors are of particular interest since practicing physicians may provide therapeutic advice if a cause-and-effect relationship is demonstrated in appropriately designed studies. Thus, smoking, an acute increase in BP and a high salt intake may increase the PWV [17, 18]. We have now studied the prevalence and correlates of arterial stiffness as assessed by PWV in patients attending outpatient nephrology clinics in order to identify modifiable risk factors for arterial stiffness that may in the future guide therapeutic decision-making. By studying nephrology outpatient clinics we obtained information that may be relevant for routine clinical practice.

## Patients and Methods

### Patients

The study was performed at Fundación Jiménez Díaz University Hospital—Autonoma University of Madrid and approved by the IIS-Fundacion Jimenez Diaz Ethics Committee. Before enrollment, the study was fully explained to all participating patients and an informed consent was signed. Patients not on dialysis attending the CKD, diabetic nephropathy and hypertension clinics were offered to participate. No limits were provided for estimated glomerular filtration rate (eGFR) or urinary albumin:creatinine ratio (UACR). Both the MDRD and CKD-EPI formula were used to for eGFR calculation, but CKD-EPI was used for statistical analyses [19]. Exclusion criteria were age under 18 years, positive serology for Hepatitis C Virus (HCV), Hepatitis B Virus surface Antigen (HbsAg) or Human Immunodeficiency Virus (HIV), being on dialysis or transplantation or refusal to sign an informed consent. This cross-sectional observational study assessed baseline data from 191 individuals. Of these, 153 were CKD patients and 38 non-CKD patients with hypertension or high cardiovascular risk. All individuals were subjected to a complete clinical history, including assessment of current pharmacological treatment, blood and urine analysis, electrocardiogram, transthoracic echocardiogram and PWV.

### Pulse wave velocity

Arterial wave reflection characteristics were assessed using the SphygmoCor CV Management System (CvMS) 2010 software version 9 (AtCor Medical, Sydney, Australia), adhering to the new EURECA-m recommendations to standardize the technique [20]. High-fidelity carotid and femoral arteries pressure waveforms were recorded by applanation ‘pencil type’ tonometry (SPT-304) of the carotid and femoral pulses. The tonometer probe is ‘applanated’ (applied so as to flatten, but not occlude an artery) at the artery. The shape of the peripheral pulse wave obtained is then calibrated with the brachial systolic and diastolic BP measured at the brachial artery) to derive the shape and dimensions of the central aortic pressure wave [21]. The best high-quality recordings; defined as an in-device quality control (operator index) of over 75%, was used for analysis. The operator index was derived from an algorithm including average pulse height variation, diastolic variation (<5%), and the maximum rate of rise of the peripheral waveform. Brachial BP was measured using an automatic sphygmomanometer. The patient was sitting or lying comfortably and allowed to rest approximately 5 minutes prior to brachial BP measurement to ensure stable hemodynamics. At least 2 minutes elapsed between brachial BP assessment and recording a pressure waveform using the tonometer. Diastolic and systolic BP, height, weight and body mass index (BMI) were entered in the corresponding fields on the Study Screen. In general, pressure waveforms were gated with simultaneous electrocardiographs recording.

Aortic (Carotid-Femoral) PWV was recorded from simultaneous measurement of the pressure wave propagation from the carotid artery (proximal) to the femoral artery (distal). Foot-to-foot PWV was calculated by determining the delay between the appearance of the pressure waveform foot in the carotid and femoral sites (Δt; transit time). The tonometry transit distance (TTD) was measured with a measuring tape on the surface of the body connecting the carotid measuring site with the femoral measuring site then multiplying the value by 0.8 [22]. Then the device software automatically estimated the carotid-femoral PWV (m/sec). PWV results were compared to the age-based reference range provided by the software and classified as above normal, high-normal, normal or below normal. A novel parameter, Delta above upper limit of normal PWV (Delta PWV**)** was defined to decrease the weight of age on PWV values. The Delta PWV was calculated as follows: Delta PWV = (measured PWV)—(Upper limit of the age-adjusted PWV values for the general population). The PWV values according to age (10-year intervals) for the general population were obtained from 16 867 subjects from 13 centers across eight European countries [9]. All patients with positive Delta PWV values were considered to have an abnormally high PWV for their age, while those with PWV within the normal limits expected for age were assigned a Delta PWV value of zero.

### Statistical Analysis

Data analyses were performed using statistical software R version 3.0.1. Quantitative variables were described by mean and standard deviation or by median and inter-quartile ranges (IQR); 25% percentile and 75% percentile. Qualitative variables were described by frequency tables and contingency tables. Differences between quantitative and qualitative variables were assessed using Student’s t test or Mann-Whitney test when comparing two groups, and ANOVA or Kruskal-Wallis test when comparing three or more groups. Correlations between quantitative variables were evaluated using Spearman correlation coefficient. In order not to overload the results section, only statistically significant univariate analysis results are presented. In order to identify potential predictors of PWV, multivariable linear regression models were fitted. Models were built using forward stepwise procedures in order to maximize R-Squared with the smallest number of predictor variables. The statistical significance of variables in the models was assessed by ANOVA test.

## Results

We performed a cross-sectional analysis of 191 patients (**S1 Table**). Most participants had DM (153/191, 80%) or CKD (153/191, 80%). This population was borderline obese with mean BMI 29.6±5.2 kg/m^2^. Mean eGFR was 64.0±21.7 ml/min/1.73 m^2^ and median (IQR) UACR 97 (23, 348) mg/g (**S2 Table**).

### Pulse wave velocity

In the whole cohort mean±SD PWV was 10.9±3.1 m/sec and 85/191 (44.5%) of patients had PWV above normal values for their age (**S1 Table and S2 Table**). PWV in CKD patients was 11.5±3.0 m/sec and for non-CKD patients PWV 8.6±2.2 m/sec. Among CKD patients 73/153 (47.7%) had mean PWV above normal values for their age. This was not significantly different from non-CKD patients (12/38, 31.6%).

There were some significant differences between patients with PWV above normal for age and other patients (**S1 Table and S2 Table**). Patients with PWV above normal for age more frequently had hypertension and DM, had higher systolic BP, pulse pressure, ejection fraction, proteinuria and uric acid levels, were more frequently on alpha blockers, and had lower urinary sodium and calcium and serum 25OHD and 1,25(OH)_2_D concentrations (**S1 Table and S2 Table**). Only 5 patients had PWV below that expected for age. These patients tended to be older (70.0±9.7 years, p = 0.082) and had a lower prevalence of DM (40%, p = 0.020) and lower aortic BP (120.2±14.7 mmHg, p = 0.018), aortic pulse pressure (47.2±11.0 mmHg, p = 0.022), UACR [48 (37, 177) mg/g, p = 0.036] and serum triglyceride levels (94±20 mg/dl, p = 0.0002) than patients with PWV above that expected for age.

We explored the association of PWV with clinical, echocardiogram, therapy and laboratory parameters (**Tables 1 and 2**).

**Table 1:** Correlations between mean PWV (m/sec) and quantitative variables in univariate analysis.

Variable	N	Coefficient	P value
Pulse Pressure (mmHg)	191	0.4692	0.0000
Age (years)	191	0.4533	0.0000
Systolic Blood Pressure (mmHg)	191	0.4207	0.0000
Serum uric acid (mg/dl)	185	0.2928	0.0001
HbA1c (%)	185	0.2588	0.0004
Ejection Fraction (%)	140	0.1964	0.0201
UACR (mg/g)	175	0.1574	0.0375
Serum glucose (mg/dl)	190	0.1542	0.0336
Serum Total cholesterol (mg/dl)	190	-0.2281	0.0015
Serum LDL cholesterol (mg/dl)	188	-0.2387	0.0010
GFR (MDRD) (ml/min/1.73 m^2^)	190	-0.2890	0.0001
GFR (CKD-EPI) (ml/min/1.73 m^2^)	190	-0.3470	<0.0001
Serum 1,25 (OH)_2_D (pg/ml)	66	-0.3363	0.0058

Only statistically significant (p <0.05) results are shown. There was a trend towards a significant correlation for serum 25(OH)D (coefficient -0.1347, p = 0.0854), serum free T3 (-0.1703, p = 0.0500) and serum potassium (0.1425, p = 0.0504).

**Table 2:** Multivariate models for predictors of mean PWV (m/sec).

	Model 1 (r^2^ = 0.3354)		Model 2 (r^2^ = 0.3114)	
	Coefficient (95% CI)	p value	Coefficient (95% CI)	p value
Intercept	-1.015 (-4.156, 2.125)	0.5243	-1.526 (-4.704, 1.652)	0.3446
Age (years)	0.062 (0.031, 0.092)	0.0001	0.063 (0.032, 0.094)	0.0001
DM	1.287 (0.296, 2.279)	0.0112	1.265 (0.252, 2.278)	0.0147
Systolic Blood Pressure (mmHg)	0.036 (0.016, 0.057)	0.0006	0.039 (0.018, 0.060)	0.0003
Serum Uric acid (mg/dl)	0.288 (0.076, 0.501)	0.0080	0.293 (0.077, 0.510)	0.0081
Calcium polystyrene sulfonate	2.968 (1.258, 4.679)	0.0008	—-	—-
Calcium-containing drugs *	—-	—-	1.640 (0.202, 3.078)	0.0256

* Calcium-containing drugs: Calcium-based phosphate binders + Calcium supplements + Calcium polystyrene sulfonate).

**Fig 1:**
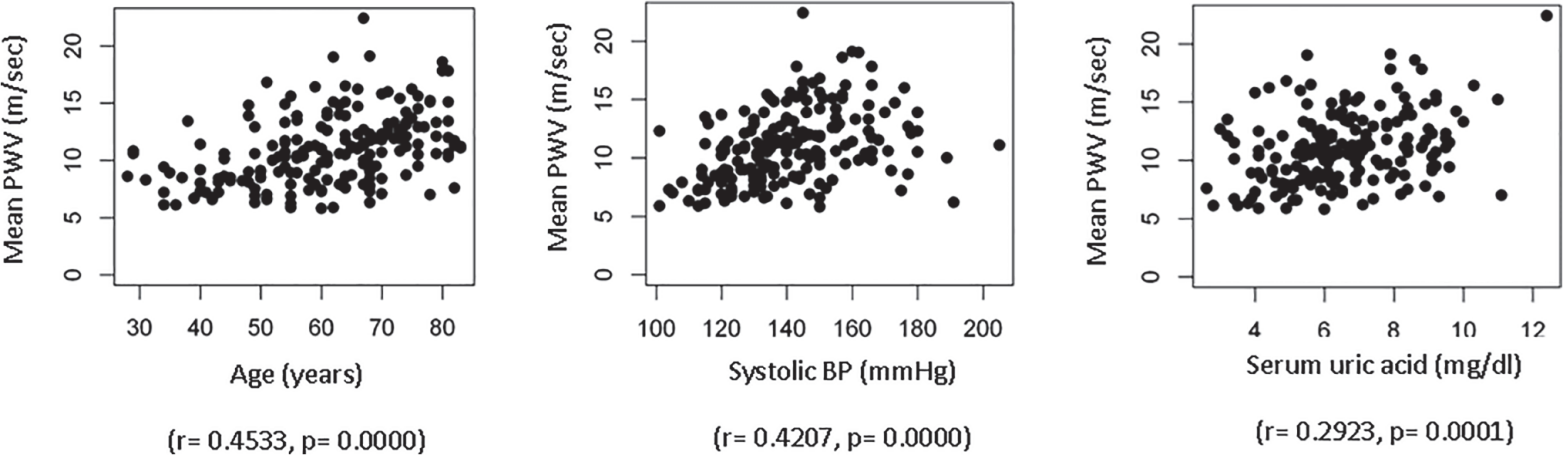
Correlations between quantitative variables and PWV that are significant in the univariate analysis and remained significant in the multivariate analysis.

PWV was significantly higher in patients with DM, hypertension, history of CVD, male gender and treated with calcium-containing phosphate binders, calcium polystyrene sulfonate or any calcium-containing medication, iron supplements, statins, ezetimibe, angiotensin receptor blockers, diuretics, alpha blockers, proton pump inhibitors or anti-platelet agents (**S3 Table**). In addition, in univariate analysis PWV (m/sec) positively correlated with age, systolic BP, pulse pressure, ejection fraction, UACR, serum uric acid, glucose and HbA1c and negatively correlated with eGFR, total serum cholesterol, LDL cholesterol and, 1,25(OH)_2_D (**Table 1**). **Fig 1** shows univariate correlations between PWV and variables that remained significant in the multivariate analysis.

Multivariate analysis (**Table 2, model 1**) showed that advanced age, systolic BP, DM, serum uric acid, and calcium polystyrene sulfonate therapy were independently and positively correlated with PWV in the best fit model. The best R squared obtained was 0.335. Age, SBP, and calcium polystyrene sulfonate therapy, but not DM and uric acid, remained independently associated with PWV after adding sex, UACR and eGFR CKD-EPI to the model (not shown). A similar model was obtained when using the variable therapeutic calcium intake (calcium-based binders plus calcium supplements plus calcium polystyrene sulfonate) (**Table 2, model 2**).

Patients on calcium polystyrene sulfonate (potassium chelation therapy) had CO_2_ 26.1±3.0 mEq/l, serum calcium 9.2±0.5 mg/dl and serum potassium 5.1±0.5 mmol/l, not significantly different from patients not on the drug (CO_2_ 28.1±3.4 mEq/l, serum calcium 9.5±0.4 mg/dl and serum potassium 4.5±0.5 mmol/l).

### Delta above upper limit of normal PWV (Delta PWV)

Since PWV increases with age and indeed we found a very significant correlation between PWV and age (**Fig 1**), we next explored parameters associated with the absolute increase in PWV over the higher expected normal limit of the age-adjusted PWV in the general population. Thus, the Delta PWV parameter was calculated as (measured PWV)—(upper limit of the age-adjusted PWV values for the general population). Mean±SD Delta PWV was 0.76±1.60 m/sec. That is, in the overall population PWV was 0.76 m/sec higher than the expected PWV according to age. For these calculations patients with no increase over age-expected values were considered to have a delta PWV of 0.00. In the patients that did have a PWV above that expected for age, the mean increase over the expected values was 2.35±2.05 m/sec. We explored the association of delta PWV with clinical, echocardiographic, therapeutic and laboratory parameters.

**Fig 2:**
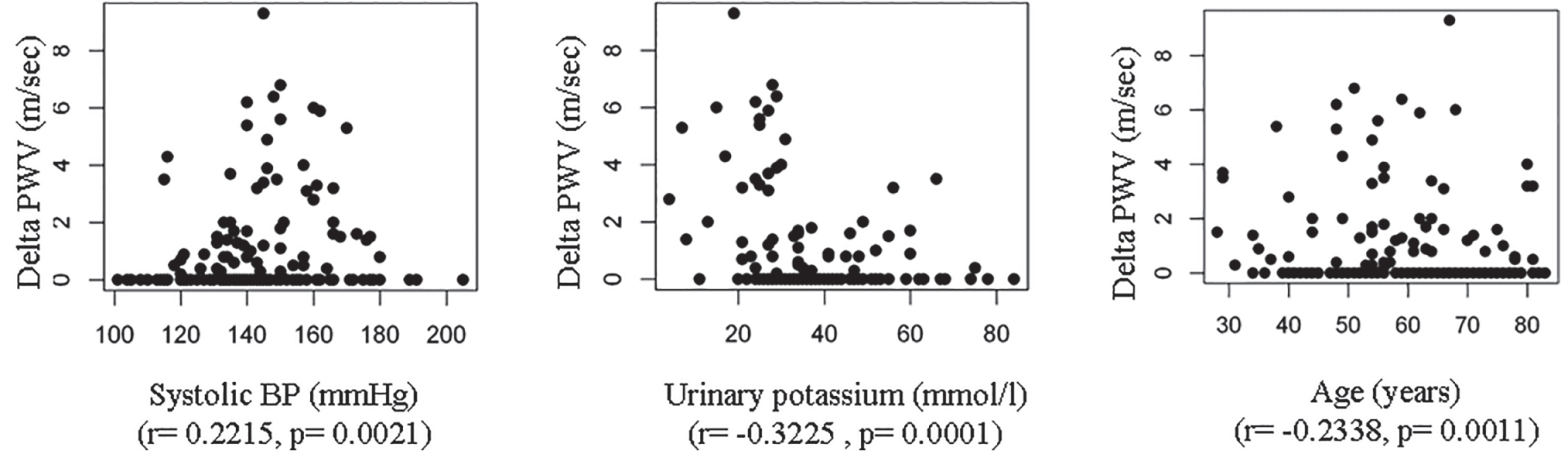
Correlations between quantitative variables and delta PWV that are significant in the univariate analysis and remained significant in the multivariate analysis.

Delta PWV was higher in patients with DM, active smoking and use of calcium polystyrene sulfonate or other calcium-containing drugs or ARBs and lower in those with valve calcification or on beta blockers (**S4 Table**). In univariate analysis delta PWV was significantly positively correlated with systolic BP, diastolic BP, mean BP, UACR and HbA1c and inversely correlated with age, serum CO_2_, serum sodium, urinary potassium and phosphaturia (**Table 3**). **Fig 2** shows univariate correlations between PWV and variables that remained significant in the multivariate analysis.

**Table 3:** Correlations between delta PWV (m/sec) and quantitative variables in univariate analysis.

Variable	N	Coefficient	P value
Urinary potassium (mmol/l)	146	- 0.3225	0.0001
Age (years)	191	- 0.2338	0.0011
Serum CO_2_ (mEq/l)	145	- 0.1986	0.0166
Urinary phosphorus (mg/dl)	131	- 0.1812	0.0384
Serum sodium (mmol/l)	189	- 0.1557	0.0324
Diastolic Blood Pressure (mmHg)	191	0.1649	0.0226
HbA1c (%)	185	0.1667	0.0233
UACR (mg/g)	175	0.1774	0.0188
Mean Blood Pressure (mmHg)	191	0.2004	0.0054
Systolic Blood Pressure (mmHg)	191	0.2215	0.0021

Only statistically significant results (p <0.05) are shown. There was a trend towards a significant correlation for serum phosphorus (coefficient 0.1395, p = 0.0618) and serum free T4 (- 0.1551, p = 0.0556).

In the multivariate analysis (**Table 4**), systolic BP, active smoking and calcium polystyrene sulfonate therapy remained independently positively correlated with delta PWV, while age, urinary potassium and beta blocker therapy were independently negatively correlated with delta PWV. The multivariate model explained 27% of the delta PWV variability. Contrary to the multivariable model obtained from PWV, the therapeutic calcium intake (calcium-based binders plus calcium supplements plus calcium polystyrene sulfonate) was not significant when used instead of the variable calcium polystyrene sulfonate (not shown).

**Table 4:** Multivariate analysis model for predictors of delta PWV (m/sec).

		Coefficient (95% CI)	p value
Intercept		0.970 (- 1.679, 3.618)	0.4703
DM		0.437 (- 0.844, 1.718)	0.5007
Gender (Male)		0.023 (- 0.626, 0.672)	0.9442
Age (years)		- 0.038 (-0.060, -0.015)	0.0014
Smoking	Active smoker	1.036 (0.331, 1.742)	0.0152
	Ex-smoker	0.409 (- 0.234, 1.053)	
Calcium polystyrene sulfonate		1.278 (0.193, 2.362)	0.0212
Beta blockers		- 0.971 (- 1.554,- 0.388)	0.0013
Systolic Blood Pressure (mmHg)		0.019 (0.005, 0.033)	0.0092
Urinary potassium (mmol/l)		- 0.029 (- 0.047,- 0.011)	0.0015

R squared = 0.2729.

## Discussion

The analysis of modifiable determinants of PWV, a measure of arterial stiffness, identified serum uric acid, UACR and calcium polystyrene sulfonate therapy or calcium-containing drugs as independently and positively correlated with PWV, while analysis of values of PWV above the age-expected normal range further identified active smoking as associated with higher delta PWV. By contrast, urinary potassium and beta blocker therapy were independently negatively correlated with delta PWV. These data identify a novel potential relationship between uric acid, calcium-containing drugs including potassium chelators and potassium metabolism with arterial stiffness.

Several aspects merit further discussion.

Stepwise multiple regression analyses revealed that the independent determinants of PWV included non-modifiable (age, presence of DM) and modifiable factors (serum uric acid, calcium polystyrene sulfonate therapy for hyperkalemia or calcium-containing drugs). Age, male gender, systolic BP, the presence of DM and UACR had been previously identified as determinants of higher PWV [23–34]. However, other modifiable risk factors were newly identified (uric acid, calcium-containing drugs including potassium chelators and potassium metabolism).

Increasing age was an independent predictor of PWV and arterial stiffness in most studies, including those enrolling dialysis and non-dialysis CKD patients [25, 35, 36]. In 1717 CKD stage 3 patients, age was the main predictor of PWV, while albuminuria was a weaker determinant and eGFR was not a determinant of PWV [25]. The increase in arterial stiffness with age is thought to be due to accumulation of abnormal collagen fibers and loss of elastin from the extracellular matrix [37, 38]. This may aggravated by hypertension that favors medial hypertrophy [12] and DM that promotes accumulation of AGE in the arterial wall [39] and the generation of reactive oxygen species that deactivate nitric oxide resulting in endothelial dysfunction [40].

There is a strong association between hyperuricemia and CV morbidity or mortality [41–49]. In our study serum uric acid level was an independent determinant of PWV. Prior studies had linked serum uric acid with arterial stiffness in diabetes and non-CKD patients. In 106 male diabetic non-CKD patients [50] and in 3772 non-diabetic non-CKD individuals [51] serum uric acid was an independent determinant of PWV and arterial stiffness. However, for the first time we link serum uric acid and PWV in a mainly diabetic CKD population. In this regard, use of allopurinol was independently associated with lower PWV in diabetic CKD patients, despite absence of significant correlation between PWV and serum uric acid levels [52].This may reflect different local practices regarding the prescription of allopurinol.

Potassium chelation therapy was an independent determinant of PWV in our study. The significance of this observation is unclear, but merits further investigation. Thus the association with potassium chelation therapy may be related to the specific characteristics of the chelation agent itself, calcium polystyrene sulfonate, a source of calcium, to potassium chelation or to the specific characteristics of the patients that lead to the need for potassium chelators (e.g. hyporeninemic hypoaldosteronism in diabetics). The observational nature of the study precludes definite conclusions as to causality. Calcium polystyrene sulfonate binds potassium, releases calcium in the gut enhancing calcium absorption, and may favor phosphate binder-induced alkalosis [53–55]. Both calcium load and alkalosis may favor vascular calcification. However, patients on potassium chelation therapy did not have higher serum CO2 nor calcium levels than the other patients. In addition, potassium chelation therapy may be a surrogate for hyperkalemia. However, patients on potassium chelation therapy did not have significantly different serum potassium than others and no correlation between the PWV and serum potassium was observed. Indeed, most patients were normokalemic. Chronic potassium chelation therapy might be associated with intracellular potassium depletion. In this regard, serum potassium levels do not always reflect intracellular potassium stores and normokalemic potassium deficiency is a risk factor for digoxin toxicity [56]. However, the nature of the data does not allow to fully supporting this concept that should be considered a hypothesis that awaits experimental validation. The need for potassium chelation therapy may also be a surrogate for medication increasing potassium levels or represent an underlying hormonal misbalance that impairs renal potassium excretion. In any case, there is some evidence for a relationship between potassium and arterial stiffness. Extracellular potassium has been reported to impact endothelial cell function and endothelial cell stiffness. An acute increase of potassium in the physiological range swells and softens the endothelial cell and increases the release of nitric oxide [57]. While a high-salt diet increases ambulatory arterial stiffness index, addition of potassium supplementation to the high-salt diet prevents the increase in ambulatory arterial stiffness index [58]. In a randomized, controlled trial a salt substitute (65% sodium chloride, 25% potassium chloride, 10% magnesium sulfate) over 12 months decreased BP and PWV compared with regular salt (100% sodium chloride) [59]. It is unclear what was driving the effect of lower PWV in this study: low dietary sodium or supplemental potassium or magnesium. In this regard, in a randomized controlled trial a higher potassium intake from fruit and vegetables or supplements did not modify PWV [60]. However, potassium supplementation has long been known to result in decreased BP in hypertensive patients with diuretic-induced hypokalemia [61] and high dietary potassium intake has a number of beneficial cardiovascular effects [62].

Potassium chelation therapy was also an independent predictor of high delta PWV and low urinary potassium was an independent predictor of high delta PWV. Information regarding the association of delta PWV with low urinary potassium does not clarify the issue but it may provide additional clues. One possibility is that low urinary potassium represents whole body potassium depletion. However, there are alternative explanations, such as an impaired ability to excrete urinary potassium or the use of potassium sparing drugs. In this regard, the impact of potassium metabolism on arterial stiffness has been poorly documented in the literature, but merits further studies.

The presence of increased PWV was not associated with LVH. As this is an observational study we can only hypothesize about potential explanations. One of them is the efficacy of current therapeutic approaches. As a representative population from Nephrology outpatient clinics in Spain, most patients were regularly followed as outpatients and 88% were receiving antihypertensive medication. Indeed, probably reflecting more difficult to control hypertension, patients with higher PWV tended to receive more frequently RAS blockade agents, alpha blockers and diuretics than those with normal PWV. Therapy may have diluted the association between PWV and LVH.

Univariate analysis disclosed some unexpected associations that were not confirmed in multivariate analyses. This includes the observation that patients receiving ARBs had higher PWV. However, use of ARBs was a marker for older patients with higher SBP and higher prevalence of DM, among others.

Delta PWV was calculated by subtracting the higher normal median PWV of the healthy general population of the same age from the patient mean PWV (i.e. it represents the difference between the expected normal PWV and the individual patient PWV when the latter was higher than expected for age). The aim of this new parameter was to decrease the impact of increasing age on higher PWV values. The use of the delta PWV variable successfully removed part of the weight of age on PWV since delta PWV was not independently associated with age in multivariate analysis. However some residual association was observed in univariate analysis. Further studies are needed to clarify this issue. This residual association may be partially explained by the fact that standard values in the general populations are available for age ranges, but not for specific ages Smoking, high systolic BP, potassium chelation therapy and low urine potassium were independent determinants of the high delta PWV, while beta blocker therapy was an independent determinant of low delta PWV. Systolic BP [23, 26, 29] and smoking had already been shown to be independent predictors of PWV in diabetic non-CKD patients [50] and in healthy individuals [63]. Beta blockers prescribed in our study were mostly vasodilating beta blockers although information on individual beta-blockers was not prospectively collected. Vasodilating beta blockers decrease PWV in humans. In a recent study, the highly selective β1-blocker nebivolol for 15 days significantly reduced PWV in 13 essential hypertension non-diabetic, non-CKD patients [64]. Nebivolol has an endothelial NO-dependent vasodilator effect [65, 66] and nebivolol, but not atenolol, had previously been associated with reduced PWV [67, 68]. In 2014 the vasodilating nonselective beta blocker carvedilol for 24 weeks was also observed to reduce PWV in hypertensive patients [69].

Among the weaknesses of this study we find that it is a single center study and, thus, the conclusions may not be extrapolated to other patient populations. In this regard, independent validation in larger multicenter cohorts would be desirable. In addition, it was an observational cross sectional study that precludes the study of causality. However, the fact that our analysis identified factors that had been previously associated with PWV supports its reliability and the potential significance of the newly identified modifiable risk factors for increased PWV that had not been previously described, such as urinary potassium and the use of potassium chelators uric acid or any calcium-containing drugs. However, we were not able to clearcut differentiate the potential contribution of potassium chelation versus calcium supplementation. In this regard, studies form countries were sodium polystyrene sulfonate is used may shed some light on the issue. The lessons on arterial stiffness and CKD from the CRIC study were recently summarized [70]. Our cohort differed from CRIC in several ways (smaller size, representative of nephrology outpatients, including those not with CKD, lower proteinuria, among others). Despite this, in both cohorts age, eGFR, DM and UACR were associated with PWV in the univariate analysis, although in CRIC UACR associated with PWV only in non-diabetics. The fact that in best fit multivariate analysis we did not observe an association between UACR or GFR and PWV may be explained by either the smaller size of our cohort or a number of differences in the characteristics of patients that include better preserved renal function, lower albuminuria, and higher usage of RAS blockade, in some patients in the form of dual RAS blockade that may improve control of UACR.

In conclusion, arterial stiffness was very prevalent in nephrology outpatients. Among modifiable factors associated with increased arterial stiffness we found systolic blood pressure, serum uric acid, potassium metabolism, therapeutic calcium and non-use of beta blockers.

## Supporting Information

S1 Table Patient characteristics, cardiovascular parameters and therapy. (DOC)

S2 Table Analytical parameters. (DOC)

S3 Table PWV (m/sec) according to categories of qualitative variables. Only statistically significant (p < 0.05) results are shown.
(DOC)

S4 Table Delta PWV (m/sec) according to categories of qualitative variables. Only statistically significant results (p < 0.05) are shown.
(DOC)

S5 Table Original database. (PDF)
