# Supplementary material for: Modifiable Risk Factors for Increased Arterial Stiffness in Outpatient Nephrology
Source: PLoS One. 2015 Apr 16;10(4):e0123903. doi: 10.1371/journal.pone.0123903 (PMC4400164; doi:10.1371/journal.pone.0123903)
Supplement: S1 Table — (DOC) [file pone.0123903.s001.doc]

**S1_Table. Patient characteristics, cardiovascular parameters and therapy**

|  | | **All**  **n= 191 (100%)** | **PWV above normal**  **n= 85 (100%)** | **PWV normal+below normal**  **n= 92 (100%)*** | **P value** |
| --- | --- | --- | --- | --- | --- |
| Age | | 61.2±12.9 | 60.0±13.3 | 61.3±13.0 | NS |
| Males (%) | | 140 (73) | 68/85 (80.0) | 62/92 (67.4) | 0.0841 |
| DM (%) | | 153 (80.1) | 75 (88.2) | 65 (73.9) | 0.0270 |
| Hypertension (%) | | 167 (87.4) | 79 (96.3) | 74 (80.4) | 0.0029 |
| CVD (%) | | 41 (21.5) | 16 (18.8) | 20 (21.7) | NS |
| CKD (%) | | 153 (80.1) | 73 (85.9) | 66 (71.7) | 0.0352 |
| Cause of CKD (%) | DM | 134 (70.2) | 67 (78.8) | 56 (60.9) | NS |
|  | Vascular | 16 (8.4) | 6 (7.1) | 8 (8.8) |  |
|  | Other | 3 (1.5) | 0 | 2 (2.4) |  |
| CKD Stages (%) | Stage 1/2  3  4-5 | 16 (8.4)/ 49 (25.7)  80 (41.9)  8 (4.3) | 9 (10.6)/ 25 (29.4)/ 37 (43.5)  4 (4.7) | 5 (5.4)/ 21 (22.8)  35 (38)  5 (5.4) | 0.0651 |
| Smoking (%) | Non | 77 (40) | 31 (36.5) | 39 (42.4) | NS |
|  | Active | 49 (26) | 25 (29.4) | 21 (22.8) |  |
|  | Ex-smoker | 65 (34) | 29 (34.1) | 32 (34.8) |  |
| Height (cm) | | 168.0±9.4 | 169.3±9.3 | 166.9±9.5 | 0.0909 |
| Weight (kg) | | 83.8±17.1 | 86.4±17.3 | 81.9±16.8 | 0.0786 |
| BMI (kg/m2) | | 29.6±5.2 | 30.2±5.8 | 29.3±4.9 | NS |
| Sys. BP (mmHg) | | 141.2±18.8 | 146.3±17.0 | 136±18.3 | 0.0001 |
| Dia. BP (mmHg) | | 78.7±12.9 | 80.1±13.3 | 77.6±12.3 | NS |
| Mean BP | | 103.1±14.0 | 106.4±13.2 | 99.8±13.2 | 0.0012 |
| Pulse Pressure | | 62.5±16.8 | 66.2±17.3 | 58.4±15.7 | 0.0018 |
| Aortic Systolic Pressure (mmHg) | | 139.9±19.3 | 144.5±17.9 | 134.9±18.6 | 0.0007 |
| Aortic Pulse Pressure | | 60.9±17.6 | 64.0±18.7 | 57±16 | 0.0090 |
| Mean PWV (m/sec) | | 10.9±3.1 | 13.2±2.7 | 8.8±1.8 | 0.0000 |
| PWV Reference Range n (%) | Below normal | 5 (2.6) | 0 | 5 (5.4) | 0.0000 |
|  | Normal | 87 (45.5) | 0 | 87 (94.6) |  |
|  | High normal | 14 (7.3) | 0 | 0 |  |
|  | Above normal | 85 (44.5) | 85 (100) | 0 |  |
| Delta above upper limit of normal PWV (m/sec) | | 0.76±1.60 | 2.35±2.05 | 0.00±0.00 | 0.0000 |
| Patients with ECHO | | 140/191 (73.3) | 64/85 (75.3) | 65/92 (70.7) |  |
| Ejection Fraction (%) | | 59.9±6.0 | 61.1±5.9 | 58.8±6.1 | 0.0361 |
| LV. Hypertrophy | | 56/140 (40) | 24/64 (37.5) | 27/65 (41.5) | NS |
| LV. Diameter (mm) | | 43.2±4.8 | 43.2±4.6 | 43.3±5.2 | NS |
| Interventricular septum (mm) | | 10.2±1.4 | 10.1±1.3 | 10.2±1.3 | NS |
| Altered Relaxation | | 91 (65) | 37/64 (57.8) | 43/65 (67.2) | NS |
| Valve Calcification | | 23 (16.4) | 12/64 (18.8) | 8/65 (12.3) | NS |
| Any vitamin D or VDRA | | 49 (25.7) | 19 (22.4) | 25 (27.2) | NS |
| Any phosphate binder | | 9 (4.7) | 5 (5.9) | 3 (3.3) | NS |
| Calcium-based phosphate binders or calcium supplements | | 8 (4.2) | 3 (3.5) | 4 (4.3) | NS |
| Calcium polystyrene sulfonate | | 9 (4.7) | 7 (8.2) | 2 (2.2) | 0.0897 |
| Calcium-based phosphate binders or calcium supplements or calcium polystyrene sulfonate | | 14 (7.3) | 8 (9.4) | 6 (6.5) | NS |
| Iron supplement | | 23 (12) | 15 (17.6) | 7 (7.6) | 0.0727 |
| ESAs | | 3 (1.6) | 2 (2.4) | 1 (1.1) | NS |
| Oral hypoglycemic agents | | 90 (47.1) | 40 (47.1) | 40 (43.5) | NS |
| Insulin | | 100 (52.4) | 54 (63.5) | 38 (41.3) | 0.0042 |
| Any lipid lowering agent | | 145 (75.9) | 70 (82.4) | 63 (68.5) | NS |
| Any anti-hypertensive ** | | 168 (88) | 81 (95.3) | 83 (90.2) | NS |
| ACEi | | 84 (44) | 39 (45.9) | 39 (42.4) | NS |
| ARBs | | 103 (53.9) | 51 (60) | 41 (44.6) | 0.0571 |
| Spironolactone | | 11 (5.8) | 3 (3.5) | 6 (6.5) | NS |
| Calcium channel blockers | | 97 (50.8) | 48 (56.5) | 44 (47.8) | NS |
| Beta blockers | | 45 (25.6) | 15 (17.6) | 26 (28.3) | NS |
| Alpha blockers | | 35 (18.3) | 21 (24.7) | 11 (12) | 0.0448 |
| Alpha & Beta Blockers | | 2 (1) | 1 (1.2) | 1 (1.1) | NS |
| Diuretics | | 107 (56) | 53 (62.4) | 46 (50) | NS |
| Proton pump inhibitors | | 69 (36.1) | 34 (40) | 29 (31.5) | NS |
| Anti-platelet agents | | 101 (52.9) | 51 (60) | 40 (43.5) | 0.0351 |

Systolic blood pressure: measured blood pressure in the peripheral arteries. Aortic Systolic pressure: blood pressure at the root of the aorta estimated from pulse wave analysis. Delta PWV= (measured PWV) – (upper limit of the age-adjusted PWV values for the general population)

* Patients with high-normal values are not included in any of the two subgroups shown.

** Including RAAS blockers, calcium channel blockers, beta blockers, alpha & beta blockers and diuretics
