# Supplementary material for: Modifiable Risk Factors for Increased Arterial Stiffness in Outpatient Nephrology
Source: PLoS One. 2015 Apr 16;10(4):e0123903. doi: 10.1371/journal.pone.0123903 (PMC4400164; doi:10.1371/journal.pone.0123903)
Supplement: S2 Table — (DOC) [file pone.0123903.s002.doc]

**S2_Table. Analytical parameters**

| Parameter (units)/ (n) | All | PWV above normal | PWV below normal+ normal* | P value |
| --- | --- | --- | --- | --- |
| Serum Creatinine (mg/dl) /(191)/(85)/(91) | 1.2±0.5 | 1.2±0.4 | 1.2±0.5 | NS |
| GFR (MDRD) (ml/min/1.73 m^2^) /(191)/(85)/(91) | 64.0±21.7 | 64.2±20.5 | 64.9±22.3 | NS |
| GFR (CKD-EPI) (ml/min/1.73 m^2^) /(191)/(85)/(91) | 65.7±24.5 | 66.0±23.0 | 67.1±25.5 | NS |
| Creatinine Clearance (ml/min) /(158)/(76)/(68) | 74.1±33.6 | 80.6±47.0 | 91.6±59.3 | NS |
| Diuresis (ml/24h) / (158)/(76)/(68) | 1894.0±584.8 | 1967.4±651.3 | 1852.1±468.3 | NS |
| Urinary Sodium (mmol/L) /(146)/(72)/(62) | 91.9±38.3 | 84.2±33.2 | 98.5±42.5 | 0.034 |
| Urinary Potassium (mmol/L) /(146)/(72)/(62) | 37.3±14.6 | 34.5±13.9 | 38.3±13.1 | NS |
| Urinary Magnesium (mg/24h) /(130)/(63)/(55) | 71.7±34.6 | 71.6±30.7 | 70.7±35.8 | NS |
| Urinary Calcium (mg/24h) /(132)/(64)/(55) | 88.9±86.1 | 70.4±66.5 | 111.9±106.8 | 0.051 |
| Phosphaturia (mg/24h) /(131)/(64)/(55) | 644.7±256.1 | 638.3±245.4 | 670.2±265.5 | NS |
| UACR (mg/g) /(175)/(82)/(79) | 97.2 (23.3, 348.0) | 112.6 (42.8, 433.7) | 71.0 (18.1, 219.0) | 0.034 |
| UACR (mg/g) <30 /30-299  /300-1000/>1000 | 68 (35.6)/ 72 (37.7)  / 33 (17.3)/ 18 (9.4) | 23 (27.1)/ 32 (37.7)  / 19 (22.4)/ 11 (12.9) | 41 (44.1)/ 35 (38.0)  / 12 (12.0)/ 4 (4.4) |  |
| Serum Glucose (mg/dl) /(140)/(85)/(91) | 140.2±56.5 | 150.7±60.8 | 133.7±53.1 | 0.039 |
| Serum Uric Acid (mg/dl) /(185)/(84)/(87) | 6.6±1.8 | 6.9±1.8 | 6.1±1.6 | 0.004 |
| Serum HbA1C (%) /(185)/(85)/(86) | 7.3±1.5 | 7.6±1.5 | 7.1±1.4 | 0.034 |
| Haemoglobin (g/dl) /(191)/(85)/(91) | 14.0±1.6 | 13.9±1.5 | 14.1±1.8 | NS |
| Serum Albumin (g/dl) /(179)/(82)/(83) | 4.3±0.3 | 4.2±0.3 | 4.3±0.2 | 0.068 |
| hsCRP (mg/dl) /(90)/(37)/(46) | 0.8±1.8 | 0.6 (0.1, 0.9) | 0.5±1.0 | NS |
| Serum Prealbumin (mg/dl) /(118)/(60)/(48) | 28.9±6.8 | 28.4±6.2 | 30.0±7.5 | NS |
| Serum Ferritin (ng/ml) /(182)/(85)/(84) | 109.5 (53.3, 192.8) | 121 (51, 188) | 106.0 (52.8, 196.3) | NS |
| Serum Total Cholesterol (mg/dl) /(191)/(85)/(91) | 163.1±37.5 | 163.3±37.3 | 164.5±38.3 | NS |
| Serum LDL Cholesterol (mg/dl) /(188)/(85)/(89) | 89.6±30.1 | 89.4±31.8 | 90.5±29.1 | NS |
| Serum HDL Cholesterol (mg/dl) /(188)/(85)/(89) | 44.3±13.2 | 43.2±13.6 | 45.2±13.1 | NS |
| Serum Triglycerides (mg/dl) /(191)/(85)/(91) | 147.6±89.1 | 157.2±97.9 | 143.2±85.8 | NS |
| Plasma Renin (ng/ml/hr) /(98)/(49)/(40) | 2.95 (1.30, 7.20) | 6.4±9.4 | 29.1±87.3 | NS |
| Plasma Aldosterone (pg/ml) / (111)/(57)/(44) | 91.0 (64.6, 129.5) | 102.4±61.0 | 107.3±70.4 | NS |
| Serum CO_2_ (mEq/L) /(145)/(67)/(64) | 28.0±3.4 | 27.6±3.3 | 28.3±3.6 | NS |
| Serum LDH (IU/l) /(188)/(48)/(90) | 374.5±89.9 | 374.7±80.2 | 379.1±98.1 | NS |
| Serum Sodium (mmol/l) /(189)/(85)/(90) | 140.2±2.7 | 139.7±2.8 | 140.4±2.5 | NS |
| Serum Potassium (mmol/l) /(189)/(85)/(90) | 4.6±0.5 | 4.6±0.5 | 4.5±0.5 | NS |
| Serum Magnesium (mg/dl) /(148)/(71)/(63) | 1.9±0.2 | 1.9±0.2 | 2.0±0.2 | NS |
| Serum Calcium (mg/dl) /(184)/(85)/(85) | 9.5±0.4 | 9.5±0.5 | 9.5±0.4 | NS |
| Serum Phosphorus (mg/dl) /(180)/(85)/(81) | 3.4±0.6 | 3.4±0.6 | 3.4±0.5 | NS |
| Serum Alkaline Phosphatase (IU/l) /(187)/(85)/(88) | 77.9±25.3 | 79.0±27.6 | 76.8±23.1 | NS |
| Serum TIBC (μg/dl) /(182)/(84)/(84) | 325.4±60.3 | 329.3±61.4 | 323.9±610 | NS |
| Serum intact PTH (pg/ml) /(164)/(80)/(70) | 51.6 (38.1, 87.1) | 68.1±48.2 | 72.0±74.9 | NS |
| Serum 25(OH)D (ng/ml) /(164)/(80)/(70) | 20.2±10.5 | 18.7±9.0 | 22.6±12.2 | 0.030 |
| Serum 1,25(OH)_2_D (pg/ml) /(66)/(28)/(32) | 34.9±14.4 | 29.8±11.8 | 38.9±16.1 | 0.017 |
| Serum Vitamin B12 (pg/ml) /(167)/(79)/(75) | 416.7±156.3 | 396.7±141.4 | 427.0±155.8 | NS |
| Serum Folic Acid (ng/ml) /(159)/(75)/(72) | 8.8±4.3 | 8.8±4.4 | 8.4±3.8 | NS |
| Serum TSH (μIU/ml) /(178)/(81)/(84) | 2.4±1.4 | 2.4±1.4 | 2.4±1.4 | NS |
| Serum Free T3 (pg/ml) /(133)/(66)/(58) | 3.1±0.5 | 3.1±0.5 | 3.1±0.5 | NS |
| Serum Free T4 (ng/dl) /(153)/(75)/(67) | 1.2±0.2 | 1.2±0.2 | 1.2±0.2 | 0.066 |
| Serum Vitamin A (mg/l) /(96)/(47)/(40) | 0.7±0.2 | 0.7±0.3 | 0.7±0.2 | NS |
| Serum Vitamin E (μg/ml) /(96)/(47)/(40) | 14.2±4.3 | 14.4±4.5 | 14.3±4.3 | NS |

* Patients with high-normal values are not included in any of the two subgroups shown.
