# Supplementary material for: Modifiable Risk Factors for Increased Arterial Stiffness in Outpatient Nephrology
Source: PLoS One. 2015 Apr 16;10(4):e0123903. doi: 10.1371/journal.pone.0123903 (PMC4400164; doi:10.1371/journal.pone.0123903)
Supplement: S3 Table — Only statistically significant (p < 0.05) results are shown. (DOC) [file pone.0123903.s003.doc]

**S3_Table.** PWV (m/sec) according to categories of qualitative variables. Only statistically significant (p < 0.05) results are shown.

| Variable | N | Mean PWV | SD | P value |
| --- | --- | --- | --- | --- |
| DM  No  Yes | 38  153 | 8.74  11.47 | 2.19  3.01 | 0.0000 |
| Hypertension  No  Yes | 24  167 | 8.36  11.3 | 2.41  2.97 | 0.0000 |
| CVD  No  Yes | 150  41 | 10.66  11.91 | 2.94  3.33 | 0.0199 |
| Gender  Females  Males | 51  140 | 9.95  11.29 | 2.85  3.07 | 0.0072 |
| Calcium Supplement or Calcium based phosphate binders  No  Yes | 183  8 | 10.85  12.84 | 3.02  3.67 | 0.0357 |
| Calcium polystyrene sulfonate  No  Yes | 182  9 | 10.72  15.11 | 2.93  2.65 | 0.0000 |
| Calcium Supplement or Calcium based phosphate binders or Calcium polystyrene sulfonate  No  Yes | 177  14 | 10.73  13.39 | 2.97  3.23 | 0.0003 |
| Iron supplement  No  Yes | 168  23 | 10.73  12.37 | 2.93  3.67 | 0.0160 |
| Statin  No  Yes | 59  132 | 9.98  11.35 | 2.79  3.09 | 0.0040 |
| Ezetimibe  No  Yes | 175  16 | 10.78  12.6 | 3.04  2.89 | 0.0221 |
| ARBs  No  Yes | 88  103 | 10.07  11.67 | 2.73  3.15 | 0.0003 |
| Alpha blockers  No  Yes | 156  35 | 10.69  12.01 | 2.98  3.21 | 0.0202 |
| Diuretics  No  Yes | 84  107 | 10.20  11.50 | 2.88  3.09 | 0.0032 |
| Proton Pump Inhibitors  No  Yes | 122  69 | 10.39  11.88 | 2.81  3.27 | 0.0011 |
| Anti-Platelet agents  No  Yes | 90  101 | 9.88  11.87 | 2.79  3.00 | 0.0000 |
