# Supplementary material for: Modifiable Risk Factors for Increased Arterial Stiffness in Outpatient Nephrology
Source: PLoS One. 2015 Apr 16;10(4):e0123903. doi: 10.1371/journal.pone.0123903 (PMC4400164; doi:10.1371/journal.pone.0123903)
Supplement: S4 Table — Only statistically significant results (p < 0.05) are shown. (DOC) [file pone.0123903.s004.doc]

**S4_Table.** Delta PWV (m/sec) according to categories of qualitative variables. Only statistically significant results (p < 0.05) are shown.

| Variable | N | Mean Delta PWV | SD | P value |
| --- | --- | --- | --- | --- |
| DM  No  Yes | 38  153 | 0.27  0.89 | 0.68  1.74 | 0.0008 |
| Smoking  Non-smoker  Active smoker  Ex-smoker | 77  49  65 | 0.43  1.36  0.72 | 0.96  2.25  1.53 | 0.0188 |
| Valve calcification  No  Yes | 117  23 | 0.91  0.38 | 1.82  0.63 | 0.0148 |
| Calcium Supplement or Calcium based phosphate binders  No  Yes | 183  8 | 0.72  1.69 | 1.55  2.50 | 0.0483 |
| Calcium polystyrene sulfonate  No  Yes | 182  9 | 0.69  2.23 | 1.54  2.18 | 0.0046 |
| Calcium Supplement or Calcium based phosphate binders or Calcium polystyrene sulfonate  No  Yes | 177  14 | 0.71  1.44 | 2.04  1.56 | 0.0520 |
| ARBs  No  Yes | 88  103 | 0.45  1.03 | 1.08  1.91 | 0.0087 |
| Beta blockers  No  Yes | 146  45 | 0.95  0.18 | 1.78  0.40 | 0.0000 |
