# Supplementary material for: Modifiable Risk Factors for Increased Arterial Stiffness in Outpatient Nephrology
Source: PLoS One. 2015 Apr 16;10(4):e0123903. doi: 10.1371/journal.pone.0123903 (PMC4400164; doi:10.1371/journal.pone.0123903)
Supplement: S5 Table — (PDF) [file pone.0123903.s005.pdf]

HTN	DM	CKD Cause	CVD	Smoking	Drinking	AGE	Gender	Height (cm)
1	1	3	1	0	0	78	1	170
1	1	1	0	2	1	60	1	169
1	0	0	0	1	0	62	2	166
1	0	2	0	0	0	82	2	138
0	0	0	0	0	0	68	2	172
1	1	1	1	0	0	70	2	156
1	1	1	1	0	0	70	1	167
1	1	1	0	1	0	74	2	150
0	0	0	0	2	0	60	2	155
0	0	0	0	0	0	68	2	152
1	1	1	0	0	0	60	2	155
1	1	2	0	1	0	64	1	154
1	1	1	0	2	0	68	1	160
1	1	1	0	1	1	67	1	174
1	1	1	1	2	0	63	2	161
1	1	1	0	0	0	76	2	156
1	1	1	0	1	0	61	1	170
1	1	1	1	2	0	68	1	175
1	0	2	1	2	1	64	1	172
1	0	0	0	2	0	64	1	170
1	1	1	0	2	1	65	1	165
1	1	1	**0**	2	0	81	1	169
0	0	0	0	0	1	55	2	170
1	1	1	0	1	0	68	2	158
1	1	1	1	0	1	76	1	167
1	1	1	1	2	0	79	1	164
1	1	1	1	0	0	81	2	154
1	1	1	0	0	0	70	2	156
0	0	0	0	0	0	68	2	165
1	1	1	0	0	1	55	1	157
1	1	1	0	0	0	73	1	175
1	1	1	1	2	0	69	1	170
1	1	1	0	2	0	68	1	171
1	1	1	0	0	0	83	2	150
1	1	1	1	2	0	67	1	180
1	1	1	0	1	1	51	1	179
1	1	1	1	2	0	83	1	167
1	1	1	0	2	0	74	1	160
1	1	1	0	2	1	51	1	181
1	1	2	1	1	0	61	1	176
1	1	1	0	2	0	82	1	161
1	1	1	1	2	2	60	1	171
1	1	1	0	2	0	61	1	176
1	1	1	0	2	0	76	1	173
1	1	1	0	0	0	76	1	169
1	1	1	0	1	0	55	1	155
1	1	1	0	2	2	67	1	170
1	1	1	0	1	1	67	1	165
1	0	2	0	1	0	51	1	170
1	1	2	1	1	1	82	1	169
1	1	1	1	2	1	72	1	168

1	1	1	0	0	0	61	2	162
1	1	1	1	1	1	81	1	190
1	1	1	1	1	1	68	1	165
0	0	0	0	0	1	58	1	167
1	1	2	0	2	0	64	1	161
1	0	0	0	0	0	68	1	167
1	1	1	0	0	0	79	2	155
1	1	1	0	0	0	74	2	150
0	0	0	0	0	0	49	2	160
1	1	1	0	0	0	66	2	155
1	1	0	0	2	1	55	1	164
1	0	0	0	2	0	59	2	155
0	0	0	0	2	0	42	1	185
0	1	1	0	2	0	34	2	160
1	1	0	0	0	0	36	1	172
1	0	0	0	0	0	42	2	167
0	1	0	0	0	0	49	1	174
0	0	0	0	2	0	58	1	157
1	1	1	0	0	0	50	2	153
1	1	1	0	0	1	41	1	180
1	0	2	0	0	2	43	1	176
0	1	0	0	0	0	39	2	160
0	0	0	0	1	0	40	2	165
1	0	3	0	0	0	81	1	181
1	1	1	1	1	0	49	1	178
1	1	1	1	2	0	55	2	161
1	1	1	0	2	0	56	1	177
0	0	0	0	1	1	50	1	174
0	0	0	0	0	0	53	1	173
1	1	1	0	2	0	59	1	175
1	0	0	0	1	0	50	1	169
1	1	1	0	1	0	59	2	166
0	0	0	0	0	0	34	1	176
0	0	0	0	0	0	40	2	163
0	1	1	0	0	1	43	1	176
1	1	1	0	1	0	48	1	177
1	0	0	0	1	0	45	1	184
1	1	1	0	0	1	47	1	180
1	0	0	0	0	0	43	1	179
1	1	1	0	0	0	45	1	162
1	1	1	**0**	2	1	48	1	180
1	1	1	0	2	0	70	2	157
1	1	1	1	0	1	73	1	162
1	1	1	0	0	0	63	2	166
1	1	1	1	0	0	73	1	172
1	1	1	1	0	1	65	2	150
1	1	1	0	1	0	66	1	174
1	1	1	0	0	0	65	1	179
1	1	1	0	2	0	67	1	172
1	1	2	1	2	0	77	1	175
1	1	1	0	1	0	67	1	165
1	0	2	0	2	1	68	1	180
1	1	1	0	0	0	79	1	168
1	1	3	1	1	1	54	1	173

1	1	1	0	0	0	56	2	154
1	1	2	1	2	1	70	1	169
1	1	1	1	2	1	71	1	173
1	0	0	0	0	0	60	1	185
1	1	1	0	1	1	60	1	173
1	1	1	1	0	0	73	1	168
1	1	1	0	1	1	71	1	175
1	1	1	0	0	0	73	1	175
1	1	0	1	0	0	76	1	171
1	1	1	0	0	0	74	1	172
1	1	1	0	1	1	71	1	168
1	1	1	0	1	0	64	1	180
1	1	1	0	2	2	72	1	180
1	1	1	0	0	0	74	2	153
1	1	1	0	0	0	69	2	155
1	1	1	0	1	1	64	1	168
1	1	1	0	2	0	75	1	160
1	1	1	0	2	1	66	1	170
1	1	1	0	0	0	68	2	161
1	1	1	1	2	0	61	1	175
1	1	2	0	2	2	67	1	175
1	1	1	0	0	0	74	1	170
1	1	1	0	0	2	60	1	161
1	1	1	0	1	0	76	1	174
1	1	1	0	2	0	44	1	180
1	0	0	0	1	1	52	1	175
0	0	0	0	0	0	31	1	172
0	1	1	0	2	0	53	1	160
1	1	2	1	1	1	54	1	190
0	1	1	0	0	0	56	2	154
1	1	1	0	2	1	57	1	167
0	0	0	0	0	0	37	2	154
1	1	1	0	0	0	78	2	157
1	1	1	0	2	0	81	1	170
1	0	0	0	0	1	40	2	174
1	1	1	1	2	1	78	1	169
1	1	1	0	0	0	54	1	158
1	1	2	0	0	1	57	1	176
1	1	1	0	0	0	64	1	155
1	1	1	1	0	0	61	2	155
0	1	1	0	2	1	73	1	167
1	1	1	0	0	0	73	2	152
1	1	1	0	2	1	35	2	162
1	1	1	0	2	1	63	1	180
1	1	1	1	2	1	76	1	157
1	1	1	0	2	1	61	1	169
1	1	1	0	1	0	58	1	170
1	1	2	1	1	0	70	1	182
1	0	0	0	2	0	52	1	170
1	0	0	0	2	0	59	1	184
1	1	1	0	0	0	34	1	177
1	1	1	1	0	1	71	1	164
1	1	1	0	0	0	44	2	155
1	0	0	0	0	0	28	1	180

1	0	0	0	2	1	54	1	172
1	1	1	0	0	0	66	2	171
1	1	1	1	2	1	75	1	176
1	1	1	0	1	2	54	1	173
1	1	1	0	0	0	63	1	170
1	1	1	0	1	1	56	1	184
1	1	1	0	0	0	62	1	168
1	1	1	0	2	1	64	1	170
1	1	0	0	2	0	44	1	165
1	1	1	0	1	0	49	2	168
1	1	1	0	0	0	40	2	147
1	1	1	0	1	1	66	1	171
1	1	1	0	2	0	81	1	175
1	1	1	0	0	0	80	2	147
1	1	1	0	2	1	54	1	168
1	0	0	0	1	1	64	1	172
1	1	1	0	1	1	29	1	169
1	1	1	0	1	1	56	1	165
1	1	1	0	1	0	29	2	160
1	1	1	0	1	0	56	1	167
1	1	1	0	0	0	80	1	156
1	1	1	0	1	1	49	1	185
1	1	1	0	2	0	54	1	170
1	1	1	0	0	0	48	1	185
1	1	1	0	1	0	38	1	169
1	1	1	1	2	0	55	1	186
1	1	2	1	1	2	62	1	179
1	1	1	0	1	1	68	1	180
0	1	1	0	2	1	48	1	168
1	1	1	0	1	0	59	1	167
1	1	1	1	2	0	51	2	170
1	1	1	1	1	1	67	1	172

Weight (kg)	BMI (kg/m2)	Sys. BP (mmHg)	Dia. BP (mmHg)	Mean BP	Pulse Pressure	Mean PWV (m/sec)	PWV Reference Range
70	24,22	123	87	101	36	7	0
95	33,26	150	78	107	72	5,8	0
75	27,22	101	65	77	36	5,9	0
56	29,41	130	73	92	57	7,6	0
60	20,28	110	62	78	48	6,3	0
77	31,64	130	60	83	70	7,1	1
81	29,04	142	78	102	64	8,4	1
54	24	120	70	91	50	8,7	1
59	24,56	104	55	71	49	7,3	1
56	24,24	115	62	80	53	7,3	1
68	28,3	152	95	122	57	7,4	1
73	30,78	136	63	84	73	7,6	1
67	26,17	150	67	98	83	7,8	1
93	30,72	108	58	80	50	7,9	1
64	24,69	120	70	91	50	7,9	1
94	38,63	158	85	113	73	9,5	1
80	27,68	143	64	98	79	8,3	1
90	29,39	132	77	99	55	8,4	1
101	34,14	114	67	83	47	8,5	1
75	25,95	130	87	101	43	8,6	1
80	29,38	132	76	96	56	8,7	1
89	31,16	146	86	113	60	10,3	1
64	22,15	113	69	84	44	5,9	1
73	29,24	127	80	96	47	9	1
82	29,3	166	82	114	84	10,5	1
74	27,51	121	58	78	63	10,6	1
50	21,08	155	56	87	99	10,6	1
69	28,35	125	69	94	56	10,7	1
71	26,08	136	72	93	64	9,2	1
70	28,40	191	99	133	92	6,2	1
83	27,1	127	82	103	45	10,8	1
90	31,14	140	59	101	81	9,5	1
90	30,78	132	80	97	52	9,6	1
72	32	158	70	99	88	11,1	1
96	29,63	125	75	99	50	9,7	1
115	35,89	133	78	106	55	6,6	1
77	27,61	142	67	95	75	11,2	1
75	29,3	134	70	94	64	11,2	1
104	31,75	134	90	113	44	6,7	1
84	27,12	166	80	110	86	9,8	1
68	26,23	141	68	96	73	11,3	1
85	29,1	162	100	122	62	9,8	1
82	26,47	150	99	122	51	9,9	1
95	31,74	122	66	94	56	11,4	1
80	28,01	140	80	105	60	11,4	1
97	40,37	147	90	115	57	6,9	1
80	27,68	147	72	96	75	10	1
87	31,96	165	87	113	78	10	1
71	24,57	105	70	88	35	7	1
77	26,96	166	86	112	80	11,7	1
79	27,99	149	74	99	75	11,9	1

74	28,2	149	81	113	68	10,4	1
99	27,42	147	72	99	75	12	1
80	29,38	134	75	100	59	10,5	1
67	24,02	123	90	101	33	7,4	1
98	37,81	180	77	117	103	10,5	1
85	30,48	140	90	107	50	10,5	1
80	33,3	127	63	84	64	12,1	1
77	34,22	178	72	114	106	12,1	1
78	30,47	121	72	88	49	6,3	1
68	28,3	135	65	95	70	11	1
82	30,49	139	85	103	54	7,9	1
66	27,47	116	65	82	51	7,9	1
158	46,7	150	99	116	51	6,6	1
54	21,09	115	72	93	43	6,1	1
111	37,52	140	84	103	56	6,1	1
120	43,03	123	83	96	40	6,7	1
82	27,08	120	68	85	52	6,9	1
68	27,59	130	83	99	47	8,4	1
76	32,47	130	85	113	45	8,5	1
83	25,62	113	63	84	50	7,2	1
124	40,03	175	120	138	55	7,2	1
112	43,75	127	66	86	61	6,7	1
88	32,32	113	72	86	41	7,3	1
80	24,42	155	88	110	67	13,4	1
102	32,19	132	85	105	47	7,5	1
78	30,09	122	68	94	54	8,9	1
92	29,37	172	104	128	68	8,9	1
61	20,15	114	74	87	40	9	1
71	23,72	121	72	88	49	9	1
87	28,41	146	92	114	54	9,1	1
79	27,66	129	84	99	45	9,1	1
63	22,86	133	88	106	45	9,1	1
87	28	123	77	92	46	7,2	1
90	33,87	132	81	98	51	8	1
88	28,41	130	80	97	50	8,1	1
94	30	154	91	119	63	8,1	1
103	30,42	123	85	98	38	8,2	1
82	25,31	144	90	117	54	8,2	1
83	25,9	144	106	119	38	8,4	1
68	25,91	120	70	93	50	8,5	1
100	30,86	126	86	101	40	9	1
70	28,4	145	78	105	67	11,8	2
77	28,96	134	72	97	62	11,9	2
64	23,23	171	79	120	92	10,6	2
72	24,34	145	94	114	51	12,2	2
53	23,56	205	110	157	95	11,1	2
90	29,73	121	65	88	56	11,1	2
88	27,46	115	65	85	50	11,2	2
106	35,83	143	83	109	60	11,3	2
92	30,04	130	50	80	80	12,9	2
69	25,34	137	66	101	71	11,5	2
93	28,7	135	89	104	46	11,7	2
79	27,99	165	80	110	85	13,3	2
104	34,75	150	80	104	70	9,4	2

62	26,14	120	70	90	50	10,2	2
89	31,16	129	60	93	69	12,3	3
84	28,07	101	49	70	52	12,3	3
95	27,76	127	93	104	34	10,9	3
91	30,41	127	87	103	40	11	3
75	26,57	136	70	96	66	12,5	3
80	26,12	155	72	101	83	12,6	3
70	22,86	138	82	105	56	12,7	3
79	27,02	130	71	91	59	12,9	3
63	21,3	142	68	99	74	13,1	3
95	33,66	140	85	108	55	13,2	3
95	29,32	150	90	117	60	11,8	3
95	29,32	130	50	77	80	13,3	3
84	35,88	161	70	101	91	13,3	3
80	33,3	150	84	112	66	11,9	3
75	26,57	143	93	116	50	12,2	3
94	36,72	120	60	83	60	13,7	3
79	27,34	165	70	116	95	12,3	3
82	31,63	180	96	136	84	12,3	3
100	32,65	154	100	124	54	12,6	3
76	24,82	177	71	113	106	12,7	3
74	25,61	155	77	108	78	14,2	3
95	36,65	156	76	118	80	12,9	3
70	22,09	165	97	131	68	14,5	3
114	35,19	136	83	101	53	8,6	3
80	26,12	189	99	129	90	10	3
137	46,31	132	63	86	69	8,3	3
85	33,2	144	92	115	52	10,3	3
97	26,87	150	100	121	50	10,3	3
66	27,83	131	76	102	55	10,4	3
110	39,44	164	100	129	64	10,4	3
120	50,6	118	72	87	46	8,5	3
77	31,24	154	60	91	94	15,1	3
91	31,49	157	78	114	79	15,1	3
110	36,33	136	90	105	46	9,2	3
85	29,76	143	71	100	72	15,2	3
71	28,44	120	70	90	50	10,7	3
95	30,67	140	85	111	55	10,8	3
82	34,13	133	61	90	72	13,9	3
78	32,47	180	95	131	85	13,9	3
99	35,5	134	61	87	73	15,4	3
87	37,66	157	61	93	96	15,4	3
64	24,39	121	74	92	47	8,9	3
113	34,88	127	80	100	47	14	3
73	29,6	141	51	93	90	15,6	3
86	30,11	150	85	110	65	14,2	3
75	25,95	139	76	99	63	11,2	3
81	24,45	145	81	111	64	15,8	3
89	30,8	131	85	100	46	11,3	3
100	29,54	137	85	102	52	11,3	3
70	22,34	134	70	101	64	9,4	3
83	30,86	176	65	102	111	16	3
70	29,14	131	80	104	51	10,1	3
153	47,22	177	89	118	88	8,6	3

87	29,4	168	111	130	57	11,5	3
100	34,2	173	73	106	100	14,7	3
96	30,99	166	72	103	94	16,2	3
96	32,08	140	87	111	53	11,7	3
60	20,76	136	98	114	38	14,8	3
112	33,08	150	89	116	61	11,8	3
66	23,38	135	75	98	60	15,1	3
85	29,41	151	76	99	75	15,1	3
80	29,38	133	90	104	43	10,6	3
106	37,56	166	102	133	64	10,6	3
55	25,45	160	90	115	70	11,4	3
78	26,67	158	82	112	76	16,2	3
74	24,16	166	66	99	100	17,8	3
61	28,23	143	52	84	91	17,8	3
80	28,34	161	91	126	70	13,3	3
94	31,77	145	91	109	54	16,5	3
113	39,56	149	88	110	61	10,6	3
88	32,32	115	80	94	35	13,5	3
65	25,39	135	83	102	52	10,8	3
78	27,97	146	96	116	50	13,9	3
79	32,46	157	68	105	89	18,6	3
84	24,54	116	72	91	44	12,9	3
124	42,91	146	77	104	69	14,9	3
107	31,26	170	100	124	70	13,9	3
66	23,11	140	90	111	50	13,4	3
83	22,99	150	88	117	62	15,6	3
67	20,91	162	88	120	74	19	3
90	27,78	160	95	123	65	19,1	3
68	24,09	140	80	106	60	14,8	3
74	26,53	148	86	111	62	16,4	3
74	25,61	150	80	108	70	16,8	3
97	32,79	145	82	108	63	22,4	3

Aortic Systolic Pressure (mmHg)	Aortic Pulse Pressure	EF (%)	LV. Hypertrophy	LV. Diameter (mm)
123	36	60	1	40
137	59	60	1	40,5
101	36	60	1	41
130	57	60	2	46,5
110	48			
		60	2	50
142	64	60	1	45
120	50	60	2	40
104	49			
115	53	60	2	34
152	57	60	2	44
136	73			
150	83			
108	50	60	2	39
120	50	50	1	47
158	73	60	2	38
143	79	60	1	42
132	55	60	2	43
107	40	65	1	47
130	43	55	2	45
132	56	60	1	47
146	60	60	2	42
113	44			
127	47	60	2	36
166	84	65	2	38
121	63	75	2	44
155	99	60	2	42
125	56	60	1	38
136	64			
191	92	45	2	41
127	45	55	1	48
140	81	50	1	49
132	52			
		60	1	45
125	50	60	1	40
133	55	60	2	49
142	75	65	2	33,5
134	64	60	2	32
134	44	60	1	44
166	86	60	1	39
141	73	60	1	45
162	62	45	1	51
150	51	60	1	53
122	56	60	2	41
140	60	60	2	50
147	57			
147	75	65	1	43
165	78	60	1	48
105	35	60	1	43
166	80	60	1	41
149	75	30	2	57,6

149	68	60	2	48
147	75	45	2	54
134	59	60	1	37
118	26			
180	103	60	1	44
140	50			
127	64			
172	99	60	1	42
121	49			
114	47			
132	42	60	2	45
116	51	70	2	33,5
115	43			
140	56	60	2	38
123	40	60	2	45
120	52			
130	47	60	2	37
130	45	60	2	41
113	50	60	2	52
175	55	50	2	50
127	61			
155	67	60	2	43,5
132	47	50	2	46
122	54	65	2	38
172	68	60	2	44
114	40			
109	37			
136	40	60	1	43
129	45			
133	45			
123	46			
132	51			
130	50	60	1	49
154	63	60	2	40
123	38			
144	54	60	2	40
144	38			
120	50			
116	28	60	2	42
145	67	60	2	37
134	62	60	2	39
171	92	60	2	41,5
145	51	60	1	44
205	95	65	1	39
121	56	55	1	44
115	50			
146	63	60	2	48
130	80	60	1	44
137	71			
135	46	60	2	42
165	85			
155	76	50	1	50

120	50	65	2	40
129	69	60	2	44,5
101	52	30	2	57
127	34			
127	40	60	1	40
136	66	55	1	44
155	83	65	2	51
138	56			
130	59	65	2	49
142	74			
140	55	65	2	41,5
150	60	60	1	48
165	102	60	2	48
161	91	60	1	42
150	66	60	2	42
143	50	70	1	40
120	60			
165	95	60	1	35
180	84			
154	54	70	1	42
177	106	70	2	43
155	78	70	2	39,5
156	80	60	2	40
165	68	70	2	43,5
121	34	60	1	43
189	90	60	2	49
119	50			
144	52			
150	50	55	2	39
111	33	60	1	43
164	64			
118	46			
154	94	70	1	42
157	79			
136	46	60	2	39
143	72	50	1	45
120	50	60	2	38
140	55	60	2	45
133	72	60	2	39
180	85	60	2	39
115	48	60	2	51
157	96	60	2	48,5
121	47			
127	47	60	1	48
141	90	70	2	40
150	65	60	2	38
139	63	60	2	41
145	64	60	2	46
131	46	60	2	41
137	52			
134	64	60	2	46
176	111	70	1	44
131	51	60	2	40
177	88			

168	57	60	2	37
173	100	60	1	47
		60	2	46
140	53			
136	38	60	2	39
150	61			
135	60	60	1	48
134	56			
133	43	65	2	36,5
166	64			
142	51			
158	76	60	1	45
166	100	60	2	43
143	91	60	1	35
153	58	60	1	42
135	42			
149	61	60	2	49
115	35	60	1	45
128	43	60	2	38
146	50	60	2	44
157	89	60	1	43
116	44	60	2	36
131	50	60	1	48
		60	2	45
140	50			
150	62	60	1	42
162	74	60	2	34
160	65	60	1	51
140	60			
148	62	55	2	46
150	70	75	2	41
145	63	70	1	50

Interventricular septum (mm)	Alterered Relaxation	Cardiomyopathy	Valvular Calcification	Pericardial Effusion
11	1	3	1	2
10,5	1	3	1	2
11,5	1	3	2	2
8	2	3	2	2
9	2	3	2	2
11,5	2	3	1	2
8	1	3	1	2
8	1	3	2	2
9,5	2	3	2	2
10	1	1	2	2
8,5	2	3	2	2
9,5	1	3	2	2
11	2	3	2	2
9,5	1	3	2	2
13	1	3	2	2
10,5	1	3	2	2
12	2	3	2	2
9	2	3	2	2
10	2	3	2	2
9,5	2	3	2	2
10	1	3	1	2
9	1	3	2	2
10	1	3	2	2
9	1	3	2	2
11,5	1	2	2	2
12	1	2	2	2
11,5	1	1	2	2
12	1	3	2	2
9	1	3	2	2
10,5	1	3	1	2
11	1	3	1	2
12	2	3	2	2
12	1	1	2	2
12	1	3	2	2
13	1	1	2	2
10,9	2	3	2	2
10	1	3	2	2
9	2	3	2	2
11,6	1	3	2	2
11	1	3	2	2
12	1	3	2	2
12	2	3	2	2
8,5	1	3	2	2

9	1	3	2	1
10,5	1	1	2	2
11,5	1	3	1	2
11	1	3	2	2
11	1	1	2	2
9	2	3	2	2
10,6	1	3	2	2
8,4	1	3	2	2
8	2	3	2	2
9	1	3	2	2
9	2	3	2	2
9	2	3	2	2
9	1	3	2	2
10,5	1	3	2	2
9,4	1	3	2	2
8	2	3	2	2
10	2	3	2	2
10	1	3	2	2
9,5	1	3	2	2
10	2	3	2	2
9,5	2	3	2	2
10	1	3	2	2
9	1	3	2	2
10	1	3	2	2
9	1	3	1	2
11	1	1	1	2
12	1	3	2	2
13	1	3	2	2
11	1	3	2	2
12	1	1	1	2
10	1	3	2	2
15	1	1	2	1

10	1	3	2	1
8	2	3	1	2
8,3	1	1	1	2
11	2	3	2	2
13	1	3	2	2
9,5	2	3	2	2
9	1	3	1	2
10	2	3	2	2
12	1	3	2	2
10,5	1	3	1	2
11	1	3	2	2
10	2	3	2	2
10	1	3	2	2
10,5	1	1	2	2
12	1	3	2	2
11,5	2	3	2	2
9,5	1	3	1	2
9	1	3	2	2
9,5	1	3	2	2
12	2	3	2	2
10	1	3	2	2
10	1	1	2	2
10	1	3	2	2
12	1	3	1	2
9	2	3	2	2
11,5	1	3	2	2
10,5	2	3	2	2
8,5	1	3	2	2
9	2	3	2	2
10	2	3	1	2
9	1	3	2	2
9	1	3	2	2
11	2	3	2	2
10	1	3	2	2
10	2	3	1	2
11	2	3	2	2
9,5	1	3	2	2
8	2	3	2	2
9,5	2	3	2	2
13	1	3	1	2
8	2	3	2	2

9	2	3	2	2
11	1	3	1	2
11	1	3	1	2
9,5	2	3	1	2
11	1	1	2	2
9,5	1	3	2	2
11	1	3	2	2
8	1	3	2	2
11	1	3	2	2
11	2	3	2	2
9	2	3	2	2
10,5	2	3	2	2
8	2	3	2	2
8	2	3	2	2
11	1	3	2	2
10,5	2	3	2	2
10	1	1	2	2
13	2	3	2	2
12,5	1	1	2	2
9	2	3	2	2
12	1	3	2	2
9	1	3	2	2
9	1	3	2	2
11	1	1	2	2

Any Vitamin D or VDRA	Any Phosphate Binders	Ca containing P-binders or Ca Supplement	Calcium Polystyrene Sulfonate (K chelator)
1	2	2	2
2	2	2	2
2	2	2	2
2	2	2	2
2	2	2	2
2	2	2	2
2	2	2	2
1	2	1	2
2	2	2	2
2	2	2	2
2	2	2	2
2	2	2	2
1	2	2	2
2	2	2	2
1	2	2	2
1	2	2	2
2	2	2	2
2	2	2	2
1	2	2	2
1	2	2	2
2	2	2	2
1	2	2	2
2	2	2	2
1	2	2	2
2	2	2	2
1	2	2	1
2	2	2	2
2	2	2	2
2	2	2	2
2	2	2	2
2	2	2	2
1	2	2	2
2	2	2	2
1	2	2	2
2	1	1	2
2	2	2	2
2	2	2	2
1	2	2	2
2	2	2	2
1	2	2	2
2	2	2	2
2	2	2	2
2	2	2	2
1	1	1	2
2	2	2	2
1	2	2	2
1	2	2	2
1	2	2	2
1	1	2	2
2	2	2	2
1	2	2	2

2	2	2	2
1	2	2	1
1	2	1	2
2	2	2	2
2	2	2	2
2	2	2	2
2	2	2	2
2	2	2	2
2	2	2	2
2	2	2	2
2	2	2	2
2	2	2	2
2	2	2	2
2	2	2	2
2	2	2	2
2	2	2	2
2	2	2	2
2	2	2	2
2	2	2	2
2	2	2	2
1	2	2	2
2	2	2	2
2	2	2	2
2	2	2	2
2	2	2	2
2	2	2	2
2	2	2	2
2	2	2	2
2	2	2	2
1	2	2	2
2	2	2	2
1	2	2	2
2	2	2	2
2	2	2	2
2	2	2	2
2	2	2	2
2	2	2	2
2	2	2	2
2	2	2	2
2	2	2	2
2	2	2	2
2	2	2	2
2	2	2	2
2	2	2	2
2	2	2	2
1	2	2	2
2	2	2	2
1	1	1	2
2	2	2	2
2	2	2	2
1	2	2	2
1	2	2	2
2	2	2	2
1	2	2	2

2	2	2	2
2	2	2	2
2	2	2	2
2	2	2	2
2	2	2	2
2	2	2	2
1	2	2	2
2	2	2	2
2	2	2	2
2	2	2	2
2	2	2	2
2	2	2	2
1	2	2	1
2	2	2	2
2	2	2	2
2	2	2	2
2	2	2	2
1	2	2	2
1	2	2	2
2	2	2	2
2	2	2	2
2	2	2	2
2	2	2	2
2	2	2	2
2	2	2	2
2	2	2	2
2	2	2	2
2	2	2	2
2	2	2	2
2	2	2	2
2	2	2	2
2	2	2	2
1	2	2	2
2	2	2	2
2	2	2	2
2	2	2	2
2	2	2	2
2	2	2	2
2	2	2	2
1	2	2	2
2	2	2	2
1	2	2	2
2	2	2	2
1	2	2	2
1	2	2	2
2	2	2	2
2	2	2	2
2	2	2	2
2	2	2	2
2	2	2	2
1	2	2	2
2	2	2	1
2	2	2	2
2	2	2	2

2	2	2	2
2	2	2	2
1	2	2	2
2	2	2	2
1	1	2	2
2	2	2	2
1	2	2	1
2	2	2	2
2	2	2	2
1	2	2	2
2	2	2	2
2	1	1	1
2	2	2	2
2	2	2	1
2	2	2	2
2	2	2	2
2	2	2	2
2	2	2	2
1	2	2	2
2	2	2	2
1	1	1	1
2	2	2	2
2	2	2	2
2	2	2	2
2	2	2	2
1	2	2	2
2	2	2	2
2	2	2	2
2	2	2	2
1	1	1	1
2	2	2	2
1	1	2	2

Any Ca. Containing Drug	Iron supplement	Erythropoietin Supplement	Oral antidiabetics	Insulin
2	2	**1**	2	2
2	1	2	2	1
2	2	2	2	2
2	2	2	2	2
2	2	2	2	2
2	2	2	2	1
2	2	2	1	1
1	2	2	2	1
2	2	2	2	2
2	2	2	2	2
2	2	2	1	2
2	2	2	1	1
2	1	2	2	2
2	2	2	1	1
2	1	2	1	1
2	2	2	1	2
2	2	2	1	2
2	2	2	1	2
2	2	2	2	2
2	2	2	2	2
2	2	2	1	2
2	2	2	1	2
2	2	2	2	2
2	2	2	2	1
2	2	2	1	1
1	2	2	2	1
2	2	2	1	2
2	2	2	1	1
2	2	2	2	2
2	2	2	1	2
2	2	2	2	1
2	2	2	2	2
2	2	2	1	2
2	2	2	2	1
1	2	2	2	2
2	2	2	2	1
2	2	2	2	1
2	2	2	2	2
2	2	2	1	1
2	2	2	1	1
2	1	2	2	1
2	2	2	2	1
2	2	2	1	1
1	2	2	1	2
2	1	2	1	2
2	2	2	1	2
2	2	2	2	1
2	2	2	2	1
2	2	2	2	2
2	2	2	1	1
2	2	2	1	1

2	2	2	1	2
1	2	2	1	1
1	2	2	2	1
2	2	2	2	2
2	2	2	1	1
2	2	2	2	2
2	2	2	1	1
2	2	2	1	1
2	2	2	2	2
2	2	2	1	2
2	2	2	2	2
2	2	2	2	2
2	2	2	2	2
2	2	2	2	1
2	2	2	1	1
2	1	2	2	2
2	2	2	1	2
2	2	2	2	2
2	2	2	1	2
2	2	2	2	1
2	2	2	2	2
2	2	2	1	2
2	2	2	2	2
2	2	2	2	2
2	2	2	1	2
2	2	2	1	2
2	2	2	1	2
2	2	2	2	2
2	2	2	2	2
2	2	2	1	1
2	2	2	2	2
2	2	2	1	2
2	2	2	2	2
2	2	2	2	2
2	2	2	1	1
2	1	2	2	1
2	2	2	2	2
2	2	2	2	1
2	2	2	1	2
2	2	2	2	1
2	2	2	2	1
2	2	2	1	2
2	2	2	1	1
2	2	2	1	1
2	2	2	2	1
2	2	2	2	1
2	2	2	1	1
1	2	2	2	1
2	2	2	1	1
2	2	2	1	2
2	1	2	1	2
2	2	2	2	2
2	2	2	1	1
2	2	2	1	2

2	2	2	1	2
2	2	2	1	2
2	1	2	2	1
2	2	2	2	2
2	2	2	1	2
2	2	2	1	1
2	2	2	1	2
2	2	2	1	1
2	2	2	2	2
2	2	2	2	1
2	2	2	1	2
2	2	2	1	1
1	2	2	1	1
2	2	2	2	1
2	2	2	2	1
2	2	2	1	1
2	2	2	1	1
2	1	2	1	2
2	2	2	1	2
2	2	2	1	1
2	1	2	1	2
2	2	2	1	1
2	2	2	2	1
2	2	2	2	2
2	2	2	1	2
2	2	2	2	2
2	2	2	2	2
2	2	2	2	1
2	2	2	1	1
2	2	2	2	1
2	2	2	2	2
2	2	2	2	2
2	1	**1**	2	1
2	1	2	1	1
2	2	2	2	2
2	1	2	1	1
2	2	2	1	2
2	2	2	1	2
2	2	2	2	1
2	2	2	1	1
2	1	2	1	2
2	2	2	2	1
2	1	2	2	1
2	2	2	2	1
2	2	2	2	1
2	2	2	1	1
2	2	2	1	1
2	2	2	1	2
2	2	2	2	2
2	2	2	2	2
2	2	**1**	2	1
1	2	2	2	1
2	2	2	1	2
2	2	2	2	2

2	2	2	2	2
2	2	2	1	1
2	2	2	2	1
2	2	2	1	1
2	2	2	2	1
2	2	2	2	1
1	1	2	1	1
2	2	2	2	1
2	2	2	1	2
2	2	2	1	1
2	1	2	1	2
1	2	2	1	2
2	2	2	1	1
1	1	2	1	1
2	2	2	2	1
2	2	2	2	2
2	2	2	2	1
2	2	2	1	2
2	1	2	2	1
2	2	2	1	1
1	1	2	2	1
2	2	2	2	1
2	2	2	1	1
2	2	2	2	1
2	2	2	2	1
2	2	2	2	1
2	2	2	2	1
2	2	2	1	2
2	2	2	2	1
1	1	2	2	1
2	1	2	2	1
2	2	2	2	1

Anti-Hyperlipidemics	Anti-Hypertensives	ACEI	ARBs	Aldactone	Ca Ch Blockers	Beta Blockers
1	1	2	1	2	2	2
1	1	2	1	2	2	2
1	1	1	2	2	2	1
2	1	2	1	2	2	1
2	2	2	2	2	2	2
1	1	2	1	1	1	2
1	1	2	1	2	1	1
1	1	1	2	2	1	2
2	2	2	2	2	2	2
2	2	2	2	2	2	2
1	1	2	1	2	1	1
1	1	1	1	2	2	1
1	1	1	2	2	1	1
1	1	2	1	2	2	2
1	1	2	1	1	1	1
1	1	1	2	2	1	2
1	1	2	1	2	1	2
1	1	1	2	2	2	2
1	1	1	2	2	1	2
2	1	1	2	1	1	2
1	1	2	1	2	2	1
1	1	2	1	2	1	2
2	2	2	2	2	2	2
1	1	2	1	2	1	2
1	1	2	1	2	1	2
1	1	2	1	2	1	2
1	1	1	2	1	1	1
1	1	2	1	2	1	2
2	2	2	2	2	2	2
2	1	1	1	2	1	1
1	1	2	1	2	2	2
1	1	2	1	2	1	1
1	1	1	2	2	1	2
1	1	1	2	2	1	2
2	1	1	2	2	1	2
1	1	2	1	2	1	1
2	1	2	1	2	2	2
1	1	1	1	2	1	1
1	1	1	2	2	2	2
1	1	2	1	2	1	1
2	1	1	2	2	1	2
1	1	1	1	2	1	1
1	1	2	1	2	1	2
1	1	1	2	2	1	2
1	1	1	1	2	2	2
1	1	1	2	2	2	1
1	1	1	2	2	1	2
1	1	2	1	2	1	2
1	1	1	2	2	1	1
2	1	1	2	2	1	1
1	1	1	2	2	1	1

1	1	2	1	2	2	2
1	1	2	1	2	1	1
1	1	2	1	2	2	1
2	2	2	2	2	2	2
1	1	2	1	2	1	1
2	2	2	2	2	2	2
1	1	2	1	2	1	2
2	1	1	2	2	2	1
2	2	2	2	2	2	2
1	1	2	1	2	2	2
1	1	1	2	2	2	1
1	1	2	1	2	1	2
2	2	2	2	2	2	2
2	1	2	1	2	2	2
1	1	1	2	2	2	2
2	2	2	2	2	2	2
2	2	2	2	2	2	2
1	2	2	2	2	2	2
1	1	1	1	2	1	2
2	1	2	1	2	2	2
1	1	1	1	1	2	1
1	2	2	2	2	2	2
2	2	2	2	2	2	2
2	1	2	2	2	1	2
1	1	1	2	2	2	2
1	1	1	2	1	1	2
1	2	2	2	2	2	2
2	2	2	2	2	2	2
2	2	2	2	2	2	2
1	1	1	2	2	1	2
1	1	1	2	2	2	2
1	1	1	2	2	1	1
2	2	2	2	2	2	2
2	2	2	2	2	2	2
1	1	1	2	2	2	2
1	1	2	1	2	1	2
1	1	1	2	2	2	2
2	1	1	1	2	2	2
1	1	1	2	2	2	2
1	1	2	1	2	2	2
2	1	1	2	2	1	2
2	1	2	1	2	2	2
1	1	1	1	2	1	1
1	1	2	1	2	1	2
1	1	1	2	2	2	2
1	1	1	1	2	2	2
1	1	2	1	1	2	2
1	1	2	1	2	1	2
1	1	2	1	2	2	2
1	1	1	2	2	2	1
1	1	2	1	2	2	2
2	1	1	1	2	1	2
1	1	2	1	1	1	1
1	1	1	2	2	2	1

1	1	2	1	2	2	2
1	1	1	2	2	1	2
1	1	1	2	2	2	1
1	2	2	2	2	2	2
1	1	2	1	2	2	2
1	1	1	2	2	1	1
1	1	1	1	2	2	1
1	1	1	2	2	2	2
1	1	2	1	2	1	1
1	1	1	2	2	2	2
2	1	1	2	2	1	1
1	1	1	1	2	1	2
1	1	2	1	2	1	2
1	1	1	2	2	2	2
2	1	2	1	2	1	2
1	1	1	1	2	1	1
1	1	1	1	2	2	2
1	1	2	1	2	1	2
1	1	2	1	2	1	2
1	1	2	1	2	2	2
1	1	1	2	2	1	2
1	1	2	1	2	1	2
1	1	2	1	2	2	2
1	1	2	1	2	1	2
1	1	1	2	2	2	2
1	1	1	2	2	1	2
2	2	2	2	2	2	2
1	1	2	1	2	2	2
1	1	1	2	2	1	1
1	1	1	2	2	2	2
2	1	1	2	2	1	2
2	2	2	2	2	2	2
1	1	2	1	2	1	1
1	1	1	2	2	1	2
2	1	1	2	2	2	2
1	1	1	1	2	1	1
2	1	1	2	2	1	2
1	1	2	1	2	2	2
1	1	2	1	2	1	2
1	1	1	2	2	1	1
1	1	1	2	1	2	1
2	1	2	1	2	1	1
1	1	2	1	2	2	2
1	1	2	1	2	1	2
1	1	2	1	2	1	2
1	1	2	1	2	1	2
1	1	1	1	2	1	2
1	1	1	1	2	1	1
1	1	2	1	2	2	2
2	2	2	2	2	2	2
1	1	1	1	2	1	2
1	1	2	2	1	1	1
1	1	2	1	2	2	2
2	1	1	2	2	2	2

2	1	1	2	2	2	2
1	1	2	1	1	2	2
1	1	2	1	2	1	1
1	1	1	1	2	1	2
1	1	1	2	2	2	2
1	1	2	2	2	1	2
1	1	2	1	2	1	2
1	1	2	1	2	2	2
1	1	1	2	2	1	2
1	1	1	2	2	1	2
1	1	2	1	2	2	2
1	1	2	1	2	1	2
2	1	1	2	2	1	2
1	1	2	1	2	1	2
1	1	2	1	2	2	2
2	1	1	2	2	2	2
1	1	2	1	2	2	2
1	1	1	2	2	2	2
1	1	1	2	2	2	2
1	1	2	1	2	2	2
1	1	2	1	2	1	2
1	1	2	1	2	2	2
1	1	2	1	2	1	2
2	1	2	1	2	2	2
2	1	2	1	2	1	2
1	1	1	1	2	1	2
1	1	2	1	2	1	2
1	1	2	1	2	1	2
1	1	2	1	2	2	2
1	1	1	1	2	1	2
1	1	1	2	2	1	2
1	1	2	1	2	1	2

Alpha Blockers	A&B Blockers	Diuretics	PPI	ASA / CLOPI / TICLOPIDINE	CKD stage	GFR (CKD-EPI)
2	2	1	1	1	3B	44
1	2	1	1	2	3B	42,9
2	2	1	2	2	0	68,7
2	2	2	1	2	3B	35
2	2	2	2	2	0	94
2	2	1	1	1	3B	41,7
2	2	1	2	1	1	90,5
2	2	1	2	2	2	63,2
2	2	2	2	2	0	99,4
2	2	2	2	2	0	89,3
1	2	2	2	2	3A	49,2
1	2	1	2	2	2	63,5
2	2	2	1	1	3B	33,3
2	2	1	2	1	1	92,4
2	2	2	1	1	3B	43,8
2	2	1	1	1	4	25,2
2	2	1	1	1	2	64,9
2	2	2	1	2	2	68,6
2	2	2	2	2	3B	32,3
2	2	1	2	2	0	89,9
1	2	1	1	1	3A	57,3
2	2	1	2	1	3B	37
2	2	2	2	2	0	83,3
2	2	1	1	1	3A	51,7
1	2	1	1	1	3A	44,6
2	2	1	2	1	3B	30,8
2	2	1	1	1	3A	53
2	2	1	2	2	3B	41,7
2	2	2	2	2	0	76
2	2	1	2	1	2	84,4
2	2	2	1	1	3B	32,2
2	2	1	2	1	3B	40,3
2	2	1	2	2	3B	51,3
2	2	1	2	2	3B	41,9
1	2	1	1	1	3A	51,6
2	2	1	2	1	2	86,8
2	2	2	2	1	3B	30
1	2	2	2	2	3A	53,8
2	2	1	2	1	2	86,8
1	2	1	1	1	4	21,4
2	2	1	1	1	3A	50,8
2	1	1	2	1	2	81,4
2	2	1	2	1	3A	58,9
2	2	2	2	2	3B	28,1
2	2	1	1	1	1	91,7
2	2	1	2	2	3B	41,4
1	2	1	2	1	4	24,4
1	2	1	2	1	4	24,4
2	2	1	2	2	4	26,1
2	2	1	1	2	2	62,2
2	2	2	1	1	3A	54,5

2	2	1	2	2	2	69,2
2	2	1	2	1	3B	32,3
2	2	1	2	1	2	87,5
2	2	2	2	2	0	93,8
2	2	1	2	1	3A	57,5
2	2	2	2	2	0	87,5
2	2	1	2	2	2	61
2	2	2	1	1	2	63,2
2	2	2	1	2	0	102,1
2	2	1	2	2	3B	33,3
2	2	2	1	1	0	95,8
2	2	1	1	1	0	100,1
2	2	2	2	2	0	110,2
2	2	2	2	2	2	96,5
2	2	2	2	2	0	96,4
2	2	2	2	2	0	91,2
2	2	2	2	2	0	104,9
2	2	2	1	1	0	93,8
2	2	2	2	2	2	86,2
2	2	2	2	2	2	82,9
2	2	1	2	2	3A	56,2
2	2	2	2	2	0	109,5
2	2	2	2	2	0	108,7
2	2	2	2	2	3A	56,4
2	2	1	2	1	2	70,6
2	2	1	2	2	2	97,9
2	2	2	1	2	3A	51,3
2	2	2	2	2	0	87,4
2	2	2	2	2	0	102
2	2	2	2	1	3A	46,5
2	2	2	1	2	0	87,4
2	2	1	1	1	2	61,8
2	2	2	2	2	0	
2	2	2	1	2	0	92,5
2	2	2	2	2	2	91,8
1	2	2	2	1	1	100,6
2	2	2	2	2	0	90,5
2	2	2	2	2	3A	59,4
2	2	2	2	2	0	81,8
2	2	2	2	2	2	72,6
2	2	2	2	2	1	100,6
2	2	2	2	1	2	74,9
1	2	1	2	2	3A	45,5
2	2	2	2	2	1	97,3
2	2	1	1	1	3B	39,1
1	2	2	2	2	3B	33,6
2	2	1	2	1	3B	41,1
2	2	1	1	1	5	10,5
2	2	1	1	1	3A	47,5
2	2	1	1	1	3A	33,3
2	2	2	1	1	1	92,4
2	2	2	2	2	3B	43,6
2	2	1	1	1	2	80,9
1	2	1	2	1	2	84,9

2	2	2	2	1	3A	50,6
2	2	2	1	1	3A	50,5
2	2	1	1	1	2	67,2
2	2	2	2	2	0	81,4
2	2	2	2	1	2	81,4
2	2	1	2	1	1	93,6
1	2	1	2	1	2	85,6
2	2	2	1	1	2	84,4
1	2	1	1	1	0	82,7
2	2	2	2	2	3A	49,1
2	2	1	1	1	2	60,5
1	2	2	1	1	3A	52,6
2	2	1	2	1	3A	42,4
1	2	1	1	1	3A	49,6
2	2	1	1	1	3B	38,4
2	2	1	1	1	2	79,2
2	2	1	1	1	3B	41,5
2	2	1	2	1	1	93,1
2	2	1	1	1	3B	35,5
1	2	1	1	1	1	91,9
1	2	1	1	1	3A	47,5
2	2	1	1	2	2	65,8
2	2	1	2	1	1	92,5
2	2	2	2	1	3B	41,2
2	2	1	2	2	1	103,5
1	2	1	2	2	0	86,2
2	2	2	2	2	0	113,4
2	2	1	2	1	2	68,6
1	2	1	2	2	2	96,5
2	2	2	2	2	1	97,2
1	2	1	2	2	2	74,1
2	2	2	2	2	0	111
1	2	1	1	1	4	23,4
2	2	2	2	1	3A	51,2
2	2	2	2	2	0	70,6
2	2	1	1	1	3A	52,3
2	2	1	2	1	2	75,7
2	2	1	1	1	2	60,6
2	2	2	2	1	2	70,6
2	2	1	1	1	2	79,8
2	2	2	1	1	3A	45,5
1	2	1	1	1	3B	34,3
2	2	2	2	2	2	95,8
1	2	1	2	1	3A	48,8
2	2	2	2	1	3A	44,6
2	2	2	2	1	3A	58,9
1	2	1	2	2	2	93,8
2	2	1	2	1	1	90,5
2	2	1	2	2	0	69,1
2	2	2	2	2	0	82
1	2	1	2	1	3B	33,9
1	2	1	1	1	3A	46,2
2	2	2	1	2	2	78
2	2	2	2	2	0	90,9

2	2	2	1	2	0	75,7
2	2	1	1	2	3A	47,2
1	2	1	2	1	2	58,8
2	2	1	2	2	2	96,5
2	2	1	1	2	3B	36,7
2	2	1	2	1	3A	55,8
1	1	1	1	1	3B	39,5
2	2	1	2	2	3B	41,7
2	2	1	2	2	0	91,1
2	2	2	2	1	3A	59,1
2	2	1	2	2	1	108,7
2	2	1	2	1	3A	52
2	2	1	1	1	3B	28,7
1	2	1	1	2	4	24,5
2	2	1	2	1	3A	52
1	2	2	2	2	0	94,4
2	2	2	2	2	2	90,2
2	2	2	1	1	1	105,5
2	2	1	2	2	3A	46,7
2	2	2	1	2	3A	55,8
2	2	1	1	1	3B	32,6
2	2	2	2	2	3A	58,6
2	2	1	2	1	3A	52
2	2	2	2	2	2	71,1
2	2	2	2	2	2	69,2
2	2	1	1	1	2	84,4
2	2	2	1	1	3A	45,5
1	2	1	2	2	3B	40,5
2	2	2	2	2	3A	64,5
2	2	2	2	1	2	73,1
1	2	1	2	1	2	74,3
2	2	1	1	1	3B	29,9

GFR (MDRD)	S. Cr	CrCl	Diuresis (ml/24h)	MAU/Cr	S. Glu	Uric Acid	HbA1C (%)	Hb
48	1,5	45	1950	25,9	151	6,2	6,1	9,3
44	1,7	62	2000	306,2	81	6	10,5	12,5
63	0,9				80	4,9	5,7	14,7
38	1,4			47,6	83	2,6		12,6
99	0,6				84		5,1	15,2
43	1,3	37	1750	15,2	168	8,2	6,8	11,5
96	0,8	87	2000	110,8	156	5,7	6,9	16,3
61	0,9	36	1600	97,6	140	5,5	8,2	11,7
102	0,6	103	1800	3,6	69	4,8	5,8	13,1
83	0,7				110	4	6,2	14,6
49	1,2	60	2200	139,5	130	6,5	6,5	15
61	1,2	91	1800	1004,7	92	5,1	6,6	12,6
35	2	31	2000	340	84	8,9	5,7	12,2
96	0,8	99	1700	23,8	74	4,9	7,9	13,1
44	1,3	66	1900	65,8	178	5,1	8,4	11,6
27	1,9	33	2400	6,1	108	9,2	8	13,5
62	1,2	84	2500	230,2	173	7,4	8,1	10,8
67	1,1	100	2800	207,8	149	6,3	6,6	14,8
34	2,1	54	1300	56,5	90	7,9	5,7	12,4
85	0,9	130	2100	4,9	102	6,9	5,7	17,3
59	1,3	89	1500	436	172	8,3	7,3	13,5
41	1,7	31	1800	15,6	129	9,1	6,1	16,7
74	0,8				84	4,1	6,2	12,8
52	1,1	81	1100	241,3	238	5,8	8	17,4
48	1,5			437,3	120	7	10	16,4
34	2	39	1500	29,2	140	5,6	6,7	11,8
57	1	34	1450	479,8	163	5,9	9,4	11,9
43	1,3	53	1700	370,7	138	9,2	8	12,8
71	0,8				85	4,5	5,9	13,8
78	1	131	1500	192,9	141	7,1	5,8	15,4
33	2	74	2500	62,9	176	4,1	8	13,5
43	1,7	47	1350	800,6	109	5,2	6,5	13,3
53	1,4	96	1400	28,1	134	7,6		16,8
43	1,2	50	1800	82,3	114	8,8	7,2	14,1
54	1,4	69	1300	66,8	107	7,8	6,1	16,2
79	1			34,8	274	5,1	7,3	15,6
39	2	42	1500	12,5	133	5,3	7,9	13,4
57	1,3	57	2900	1085,1	136	7,2	7	16
79	1	94	1800	239	169	7,4	8,2	14,1
23	3	27	1600	27,6	108	6,9	10,7	15,3
56	1,3	40	1950	124,2	79	6	6,8	13,4
76	1	81	2800	155,1	187	6,1	9,5	15,4
56	1,3	89	1500	112,3	158	8,2	7,5	12,8
33	2,2	44	2000	312,4	114	6,5	7,4	16,3
110	0,7	169	1800	199,2	146	5,4	7,1	13,2
42	1,8	53	1450	1686,6	110	9,3	6,2	16,6
26	2,6	42	2550	1261,2	168	6,9	7,1	12,8
26	2,6	33	1300	57,1	158	6	8,4	11,9
27	2,7	37	1700	27,2	95	11,1	5,6	12,2
64	1,1	45	1750	1,1	139	6	7,8	11,9
58	1,3	81	2250	271,5	104	6,2	8,7	14,7

64	0,9	122	2000	164,7	89	6	5,8	14,3
36	1,9	45	1450	91,6	150	6,1	7,4	12,9
84	0,9	122	1700	71	218	5,6	8,7	14,1
87	0,9				78	5,9	5,5	15,3
59	1,3	79	1850	20,6	206	6,7	7,6	14,1
96	0,9			4,4	109	5,9	6,4	17
60	0,9	108	1900	93,5	130	4,8	6,8	14,2
61	0,9	93	1950	24,9	156	3,2	8,9	12,5
82	0,7				94	3,8	5,5	12,4
34	1,6	53	1400	3,8	70	5,7	6,2	9,3
88	0,9	98	600	2,8	105	6,3	6,6	13,7
102	0,6			5,4	94	5,6	5,8	14
106	0,8			4,8	94	5,2	5,3	15,5
82	0,8	118	1300	9,6	291	2,8	8,2	13
85	1			1,2	168	3,5	7,3	16,4
79	0,8			6,2	124	3,9	5,6	14,8
105	0,8				88	4,6	4,9	14,5
87	0,9				92	5,6	6	15,9
76	0,8	125	1600	142	195	4,2	9,4	12,9
74	1,1	112	1440	4,2	153	6,5	7	14
54	1,5	77	2100	135	249	6,5		17,7
93	0,7			3,1	128	3,4	7	13,6
93	0,7				76			14
58	1,2	73	2200	2,3	90	7,1	5,9	15,2
64	1,2	153	1800	121,5	160	8,4	8,4	13,7
87	0,7	108	3000	941,4	254	4,1	7,4	15,4
51	1,5	64	1700	813,2	122	8	6,7	15,5
79	1				78		5,4	14,9
101	0,8	103	1200		96	4,5		14,7
47	1,6	20	1900	37,2	94	9,1	5,5	14,1
79	1	59	2000	168,4	122	7	5,5	17,5
60	1	47	1900	77,8	112	5,4	5,9	15,5
79	0,8			463,9	103	4,8	5,7	14,4
82	1	98	1900	51,8	131	4,6	7,1	15,5
90	0,9	110	2500	627	333	6,2	12,8	12,1
81	1			2,5	98	6,9	5,8	14,3
58	1,4	75	2400	43,1	177	6,9	8,2	15
73	1,1			4,7	82		5,9	15,9
65	1,2	71	3000	41,2	249	5,9	6,9	14,9
90	0,9	55	1600	110,9	76	6,1	9,1	16,1
71	0,8	112	1100	75,2	128	7,2	6,8	15,2
49	1,5	57	2300	97,2	137	9,5	8,3	14,2
101	0,6	81	1150	520,9	82	5,2	7,1	12,1
42	1,7	29	2000	17,1	104	6,2	6,3	12,5
34	1,6	40	900	1782,4	134	8,4	7,3	14,2
43	1,7	43	1500	312,2	168	9,5	7,6	16
12	5,3	35	2800	1360,3	59	8,3	6,2	10,9
47	1,5	75	2850	7,1	87	7,3	9,4	13,7
45	1,9	82	2450	3,4	174	7,4	7	12,9
96	0,8	111	1150	114,9	159	3,4	7	12,7
46	1,6	69	2000	82,1	99	8,9	5,7	16,7
81	0,9	73	950	220,8	74	5,4	6,7	14,4
78	1	114	1300	2077,2	97	9,6	6,7	14,4

49	1,2	40	1350	1,8	171	4,6	7,6	13,2
53	1,4	75	1850	22,8	154	5,9	8,1	13,9
66	1,1	93	2500	56,2	82	9,5	6,9	11,7
76	1			3,6	105	7,1	6,2	14,6
76	1	104	1750	274,4	143	6,7	6,5	15,7
111	0,7	133	1700	47,1	97	4,1	7,9	14,5
88	0,9	6,2	1800	1476,1	153	5,9	6,2	14,5
84	0,9	105	1400	98,1	203	3	8,5	13,7
82	0,9			4	127	7,8	6,1	14,5
53	1,4	68	1950	497,1	99	5,6	7,3	12,7
60	1,2	96	2650	78,6	250	7,8	10	14,6
59	1,3	43	1800	1026,5	108	7,8	7,4	14
45	1,6	57	1600	153,8	285	8,1	9	13,1
52	1,1	55	1950	45	143	6,7	7,3	11,7
40	1,4	42	1500	43,4	221	8,4	8,7	14,3
75	1	123	3500	42,6	160	7,2	7	16,7
45	1,6	60	1700	226,2	103	6,8	6,8	15,4
97	0,8	150	2700	262,1	111	5,8	6,1	13,8
37	1,5	70	1800	46,1	238	9,1	8	12,6
92	0,9	129	2300	226,3	237	6,9	8,6	14,8
50	1,5	57	2000	15,5	174	9	6,4	12,1
60	1,1	54	1000	113,1	231	9,8	8,5	12,4
**91**	0,9	103	2000	53,4	97	7,8	8,3	14,5
45	1,6	30	1500	648,7	132	8,4	6,5	15,2
92	0,9	167	3660	25,1	330	5,8	11,8	16,4
78	1			51,8	95	7,5	5,6	13,9
98	0,9				93	5,3	6,1	13,1
63	1,2	113	2800	2166,5	239	6,2	11,4	14,4
88	0,9	99	1800	235,3	172	5,4	8,2	15,8
87	0,7	101	1500	82,2	258	4,1	11,9	13,3
69	1,1	62	1900	3745,5	79	7	4,8	13,7
94	0,7			5	82	5,9	5,9	14
26	2	21	1200	276,4	86		6,9	10,8
56	1,3	53	1050	2,7	157	6,6	5,9	13,6
61	1				78	4,9	5,6	13,5
57	1,3	62	2260	356	113	11	7,9	13,5
70	1,1	55	1700	195,9	119	8,4	6,8	13,5
60	1,3	79	2000	3,5	157	5,9	7,6	14,3
53	1,1	68	1450	12	133	6,4	6	12,6
73	0,8	95	1500	1578	52	8,5	8,2	13,2
49	1,5	63	1900	85	171	8,3	7,1	11,7
36	1,5	32	1300	15,7	118	7	7,6	12,8
82	0,8	85	1000	447	152	3,9	9,3	11,5
50	1,5	54	1900	109,8	175	6,2	6,9	12,9
48	1,5	53	1550	16,4	170	9,2	7,6	14,4
60	1,3	88	1000	97,9	107	6,5	7,1	14,6
87	0,9	95	2000	140,1	178	5,4	7,6	14,9
96	0,8	89	2700	374,2	86	4	5,5	14,6
64	1,2	79	1700	1,9	108	7	6	13,1
76	1				82	6,8	5,2	16,9
33	2,4	32	1950	2097,3	208	9,1	9,9	13,4
49	1,5	47	3400	740,5	152	5,3	6,5	10,7
78	0,9	48	1700	238,4	89	3,4	6,4	13,8
80	1,1			9,6	119	6,4	6,7	15,2

70	1,1	152	1650	56,3	126	9,6	6	15,5
48	1,2	77	2000	782,5	164	7,6	8,3	10,7
60	1,2	52	1800	87,7	169	8,1	8	14,3
88	0,9	89	1550	334,3	161	5,9	7,7	16,8
37	1,9	42	1200	16,8	147	8,9	7,9	14,3
56	1,4	68	2000	54,5	183	5,9	9,3	14,5
41	1,8	68	3200	1514	137	9,2	6,3	11,6
43	1,7	84	3800	53,7	91	6,9	7,4	13,2
81	1	56	1650	48,8	157	6,7	7,6	14,6
56	1,1	103	1150	401,3	79	6,2	5,8	12,7
93	0,7	83	2850	17,1	114	4,4	6,5	10,9
54	1,4	59	2000	2768,2	137	4,4	6	11,9
32	2,1	46	1550	2,2	132	7,9	9,9	13
27	1,9	30	1000	436	95	8,8	7,5	13,9
52	1,5	51	2000	507,2	138	10	8,4	14,7
97	0,8			19,9	75	5,6	5,6	15,9
88	1,1	172	1800	317,3	225	6,4	9,8	13,5
117	0,7	141	850	112	208	3,2	8	16,2
43	1,5	43	2800	426,6	152	6,3	7,1	13,1
56	1,4	114	2000	456,3	166	6,2	8,6	15,8
36	1,9	38	1300	948	367	8,6	10,1	15
57	1,4	96	1650	20,5	94	5,6	6,6	12,7
52	1,5	97	2350	15,4	249	6,2	8,5	14,1
65	1,2	52	2650	425,2	67	8,3	4,8	13,5
62	1,3	54	2400	74,4	235	6,6	9	15,3
78	1	132	3200	3367,7	191	6,6	6,9	15,9
47	1,6	60	1750	2,8	211	5,5	8,9	15
43	1,7	24	1700	1438,3	175	7,9	7,8	11,8
60	1,3	36	2400	2000	134	5,5	9,7	14
73	1,1	81	2400	565,4	83	10,3	8	17,3
66	0,9	72	2000	390,8	174	4,9	9,5	12,7
32	2,2	52	3000	301,6	163	12,4	9	12,3

Albumin	hs-CRP	Pre- albumin	Ferritin	Total Cholesterol	LDL Cholesterol	HDL Cholesterol
3,8	7,05	22,3	509	153	75	60
3,9	0,34	30,9	73	172	97	50
4,1			28	139	72	52
4,3	0,86		198	187	123	45
4,2	0,03		42	231	81	73
4,3	1,26	23,2	88	197	94	42
4,3		27,2	80	124	27	34
4,2			76	141	72	52
4,4	<0.5		153	187	121	56
	0,13		89	173	96	56
4,9			70	210	120	47
4,1			22	114	61	31
4,3	1,06	26,6	41	115	67	28
4,3	0,8	14,2	369	139	88	24
4,2	0,08	35,9	54	125	60	30
4,1		33,9	407	197	102	47
4,1	0,75	27	80	138	86	38
4,7		42,8	139	200	113	49
4,2			281	148	82	50
4,5	0,16		392	186	114	53
3,7		28,9	152	154	104	24
4,1		20	199	145	79	46
4,2	0,3		58	217	139	65
4,5				186	59	45
			24	114	56	36
4,3		27,4	1215	159	87	49
4,5	0,01	36,6	423	148	81	33
3,9		36,3	32	168	103	44
	0,1		77	229	139	68
4,6		32,2	333	147	112	29
4,3	0,6	27,2	223	127	75	36
4,4		22,8	64	136	77	40
4,1			126	224	119	55
4,7	0,03	31,4	128	178	133	70
4,6	0,11	33,5	46	134	79	41
4,1			91	165	104	41
4,4		17,7	388	170	76	33
4,1	0,25		149	192	118	47
4,2	0,8	50,9	105	223	120	32
4,5	0,42	30,2	148	188	100	36
4,1	0,04	24	35	147	75	49
4			107	174	85	53
4,3		32,2	197	139	75	38
4,3	<0.5	41,5	19	126	67	37
4,4	0,3	22,5	12	204	144	42
4,2		43,2	243	193	112	42
4,2	0,03	35,5	124	108	48	46
4,4			180	155	83	37
4,7		38,5	173	120	43	30
4,1		22,7	78	118	62	40
4,2	0,13	28,9	29	146	101	46

4,3	0,39	32,6	115	137	74	47
4,4			49	126	52	33
4,3		27,9	6	109	55	26
4,2	0,17		54	250	169	44
4,5		29,7	59	158	67	57
4,4	0,04		306	144	81	41
4,3		18,5	98	156	78	58
4,3		21,6	10	69	26	33
	0,57		28	182	95	41
4,2		24,9	250	150	92	34
4,4	0,03		10	121	59	34
4,2	0,07		119	159	106	36
3,7	0,91			185	111	66
4,1		20,8	27	175	80	84
4,7	0,25	29,8	136	145	77	36
4,4	0,92	24		142	62	53
			492	153	81	62
4,6	0,86		196	304	144	50
4,1	0,08		193	189	83	35
4,3		30,6	59	196	89	97
4,5				138	59	48
4,2	0,6		29	204	145	35
			129	216		
4,5	0,04		65	116	51	47
4,6		37	265	149	112	47
5	0,89		95	134	79	31
4,5		35,4	312	153	101	43
	0,03			190	97	79
4,2	0,01			136		
4,1	0,1	32,1	160	117	54	40
4,4	0,66		22	203	70	49
4,6			25	184	90	37
4,2	0,39		20	177	111	35
4,6	0,08	33,8	148	147	77	50
3,7			96	160	115	44
4,5	0,41		148	194	123	51
4,2		41,9	420	156	96	33
	1,08			264	189	41
4,4			108	196	100	46
4,6		29,9	294	171	95	55
4,4	0,7	28,5	227	175	96	58
4,2		34,6	110	147	77	37
3,9		24,1	83	121	63	49
4,6			195	126	78	55
3,9		12,4	623	227	138	57
4,4	0,29	24	194	147	94	30
3,9			97	146	83	39
4,3	0,12	29,2	24	129	66	33
4,2		26,1	57	152	89	35
4	0,42		6	129	50	33
4,4	0,2			224	150	45
4,2		30,9	71	114	61	38
4,3	0,04	33	64	155	63	68

4,3	0,01	26,8	64	151	84	46
4,1	2,2	27	28	191	86	77
4,4	0,7		103	101	52	39
4,2	0,08		322	248	126	64
4,1		30,6	188	170	62	38
4,1			15	125	76	39
3,9		26,7	126	135	75	33
3,8	0		192	91	49	27
4,4	0,01		138	106	52	38
4,5	0,71	29,3	398	141	77	44
4		21,6	257	172	80	38
4,3	0,24	35,5	173	174	73	33
4,3	0,21	34,4	209	132	59	32
4,2		29,6	78	154	74	51
4,5	0,64	22,2	34	242	153	43
4,6		30,1	127	147	76	50
4,2	13,99		66	171	95	47
4,4	0,83	33,9	53	137	77	43
4,3		27,8	232	162	98	41
4,3		20,2	121	107	31	19
4,3	0,63	34,2	82	132	58	42
4,6			9	223	101	33
4,3		27	265	166	99	43
4,5		33	54	219	128	37
4,3	0,1		612	237	165	22
4,4	0,95		92	238	154	48
4,3	0,81	21,6	49	122	70	39
3,8	1,57	34,3	185	190	75	36
4,6		31,5	336	141	79	28
4,2		25,5	358	173	102	51
2,8		23,4	123	226	152	58
4,2	3,08		56	167	68	49
	0,77		124	165	76	45
4,1			34	169	120	34
4,5		29,8	81	223	138	69
4,6		27,1	24	135	70	30
4,3			105	151	78	56
4,1		28,2	261	157	109	32
4,2	0,81	21,5	75	142	77	42
3,6			87	145	82	45
4,8	0,76	29,4	74	150	108	39
4,2	0,01		14	138	73	40
3,8	1,42	21,4	21	221	186	64
4,1		29,9	327	166	72	63
4,3	1,71	26,4	43	123	61	36
4,6		33,5	428	149	84	42
4,3		27,8	166	115	72	34
4,6		26,8	27	168	118	35
	0,1		537	188	115	38
	0,09		148	205	144	36
4,3	0,06	29,4	143	200	131	46
4,1			137	134	56	61
4,3		29,5	16	164	97	49
4,4	0,88		46	159	104	31

4,9	0,17		234	274	194	52
4,2	1,01		117	166	93	33
4	0,52		112	126	78	32
4,4		25,06	125	152	81	28
4,4		30,7	44	108	47	24
4,2		22,8	135	189	105	40
3,7	0,01	49,9	280	172	100	42
4,4		25,6	191	152	94	47
4,6			126	180	69	27
4,1			49	147	92	36
4,2	0,06	26,7	3	140	71	43
3,8		32,7	244	105	48	32
4,3	0,02	40	133	110	49	39
4,2		30,8	69	198	106	41
4		37,8	161	134	66	40
4,3	0,07	29,4	716	179	116	42
4,1		19,2	119	207	103	39
4,3	0,26	22,8	109	179	110	39
4,3		23,3	27	154	73	60
4,6		33,3	34	177	68	35
4,2		24,2	32	139	43	41
4,4		33,4	85	166	97	57
4,3		29,2	177	164	78	39
4,5	7,3	12,3	94	169	118	43
4,4		43,3	167	201	88	101
3,4	0,05	24	261	133	87	47
4,5		31,7	44	180	83	64
4,4		22	179	138	53	33
3,5		28,7	121	191	100	71
3,3			23	182	71	49
3,7		16,5	51	159	70	81
4,3	>1.5	29,9	206	105	52	29

Triglicerids	Renin	Aldosterone	CO2	LDH	Na	Nau (mEq/L)	K
91			24	355	140	62	3,9
127	252	222	27	525	140	105	5
75				336	141		3,8
97			27	389	135		4,9
81				285	141		4,4
154	25,8	278	29	555	135	82	5,3
206	<0.2	114	34	347	141	50	4
71			22	315	139	44	4,5
50			33	457	140	78	3,7
104				345	143		4,2
215			29	391	141	57	4,8
92			33	405	141	119	3,5
228			27	332	139	32	5
135	1,6	40,3	23,4	335	138	49	4,7
184	0,2	113	28	428	139	100	4,2
242	4,2	144	26	463	141	55	4,5
71	3,5	113	27	326	140	121	4,9
191	0,7	59,7	33	347	141	125	4,4
79			24	355	137	74	5
94				311	145		4,3
129	0,2	101	30	612	139	143	4
102	7	118	33	423	143	108	4,4
64				425	140		4,1
409			22	533	138	167	4,7
112				346	146		5,4
114			23	366	141	101	5
168	1,5	118	29	334	146	54	5
103	9,7	333	25	544	138	37	4,8
109				407	142		4,2
191	<0.2	111	28	321	143	124	4,3
135			33	318	142	128	5,2
95	0,3	157	30	360	143	84	4,6
185				412	140	168	4,2
155	5,1	65,8	27	379	145	114	5,5
70	0,8	91	28	293	143	105	4,7
99			27	428	139		5
160	5,2	121	29	341	143	143	4,4
134			35	402	143	47	4,5
292	5,7	103	37	409	138	81	4,9
262		97,7	28	437	137	58	4,9
50	15,8	111	32	438	141	41	5,1
179	9,2	87,5	28	396	141	101	4,6
101	34,1	25	28	296	142	122	4,3
111	60	122	25	473	142	97	5,1
89	36,7	71,7	29	560	145	145	4,3
197	1,4	134	30	337	142	147	4,2
72	1	47,6	22	279	139	224	5
212			32	350	136	71	4,1
159			22	425	138	84	3,7
78	2,7	86,7	23	262	136	68	4,5
83	12,2	93,3	27	377	142	103	4,1

78	500	341	33	282	144	114	4,3
169			25	318	141	101	5
255	44,7	33	30	393	140	147	5,7
183				390	141		4,5
170	1,4	68,7	29	427	141	105	4,6
111				349	142		4,1
82	0,7	95	34	301	141	104	3,6
51	0,8	47,9	26	393	138	133	4,7
228				404	141		3,8
119	2,8	38,4	27	453	140	162	4,8
141			32	380	140	219	4,4
87				346	144		4,1
41				358	141		4,4
53	0,6	17,1	26	356	137	87	4,6
165				300	140		4,2
133				321	141		4,4
49				285	141		4,5
550				369	137		4,4
357	0,5	68	23	307	135	135	4,6
48	0,5	57,8	32	350	137	70	4,6
153				414	139	70	5
121				301	139		4,6
195							
92				305	141		4,3
164	90,3	70	28	174	144	118	4,2
120			30	358	138	130	3,7
157	15,4	56,6	28	367	140	67	4,9
68				269	141		4,4
52				279	144		4,3
114	<0.2	148	22	316	139	18	3,9
420			32	977	142		4,5
285	0,3	86	31	341	142	110	4,1
157				302	142		4,5
99	2,3	56,6	30	528	137	72	4,3
134				439	137		4,8
99				313	142		4,2
137	6,7	155	29	349	139	51	4,7
171				324	142		4,8
112			28	369	137		5,7
107	1,3	101	28	426	138	73	4,8
107	1,4	55,1	22	444	140	163	4,7
163	0,7	263	31	422	144	75	3,8
45	7,5	44,8	30	411	144	145	4,6
91			26	184	139	24	4,7
158	2,8	51,1	25	364	142	123	5,1
113	2	177	28	373	141	101	4
120			27	451	138		5,2
152	2,9	68,2	29	262	139	67	5,5
140	2,5	324	27	311	137	92	4,6
127			32	265	139	130	4,2
143			29	354	146		4,3
73	1,2	153	27	187	145	86	3,6
122		51,2	32	439	141	124	5,1

105	20,1	93,8	27	338	141	118	4,8
142	3	53,8	35	305	142	89	4,3
52			32	429	140	85	5,2
289			30	386	139		5,6
221	41,1	89	26	353	142	114	5,5
50				274	141	97	4,3
100	7,3	93,6	32	378	142	49	5,3
73			30	353	139		5
79				309	138		3,7
90			33	312	141	62	4,8
270	0,2	197	27	368	142	53	4,3
339	2,3	74,2	27	262	139	45	4
207			32	407	137	52	5,5
145	1,5	34,4	28	398	142	87	4,9
230	3,7	72,4	28	366	142	88	4,5
106	>29.3	164	24	468	138	54	4,1
144	1,6	65,6	28	325	143	129	4,6
87	0,9	33,2	28	402	138	111	4,6
115	1,4	173	29	366	140	102	4,3
448	0,6	131	30	336	138	98	3,9
161	3,6	99,4	27	297	141	93	4,5
443			25	403	137	125	4,5
121	11,5	104	28	420	144	93	4,5
272	1,1	60	30	344	141	56	4,7
138				315	135	123	4,5
181				363	139		4,5
64				396	139		4,2
394	1,4	101	26	415	142	71	4,1
171	<0.2	71,3	31	354	143	121	4,1
98	0,4	162	28	470	139	151	4,6
78			21	478	142	118	5,6
250				370	138		5
219				463	147		4,7
144			28	333	141	135	5
79				314	140		3,9
177	1,1	71,2	28	377	144	37	4,5
86	3,7		30	347	141	84	5,3
82	2,1	75,7	29	288	138	88	4,4
116	6,7	69,3	31	413	138	74	4,8
92			26	618	142	100	4,7
162	1,9	71,8	28	384	141	68	4,1
125	8,7	130	29	424	142	99	4,3
116	40,5	46	26	266	137	83	4,4
156			27	810	139	55	5
130	>31.7	71	22	248	134	62	3,9
113	2,7	84	24	366	144	154	4,9
45	2,4	43,4	24	303	140	104	4,6
89	<0.2	70,9	33	329	143	97	3,8
173				322	139	79	4
123				328	141		4,3
114		175	30	252	138	47	4,5
86	0,5	150		383	134	33	5
90	6,6	293	25	261	140	69	4,8
121				531	139		4,4

142				387	142	91	4,9
198				369	142	101	4,6
82	16	31	34	311	140	28	4,8
214	1,12	134	27	301	138	155	4,2
82	4	129	30	325	146	94	5,2
218	5	80,3	28	406	139	87	5,1
149	34	120	26	416	141	44	4,3
118	13,3	103	27	333	141	71	4,6
418	>33.5	61,6		421	136		4,5
97					140		4,5
131	8	374	24	338	134	14	4,9
126	0,8	61,5	25	322	140	81	5,7
137	6,1	63,9	26	468	141	112	4,8
254	5,4	152	24	405	140	79	5,9
140	>29.6	111	27	309	137	83	4,4
103				500	144		4,2
324	1,6	65,2	30	387	137	80	4,7
151	3	88,7	26	355	135	164	4,4
104	3,5	34	27	393	139	84	5,3
497	3,6	51,2	30	427	141	101	4,6
274	5,9	103	28	396	142	143	4,7
60	1,2	67	31	340	141	89	4,1
235	2,7	102	21	318	134	124	4,8
40	6,5	165	20	318	142	21	3,5
59	>25.2	35,5	32	382	139	40	4,4
94		91	25	425	136	98	4
105	>32.2	134	28	362	138	58	5,4
260	3,2	128	23	379	134	51	5,1
102	2,2		33	419	139	83	4,8
88				386	143	61	4,7
40	3,1	80,5	25	451	137	37	5,2
120	22,5	138	20	346	135	51	4,1

Ku (mEq/L)	Mg	Mgu (mg/dl)	Mgu (mg/24h)	Ca	Cau (mg/dl)	P	Pu (mg/dl)	Pu (mg/24h)
39	1,86	2	39	8,9	3,5	2,4	30	585
34	2,03	5,6	112	10,1	4,2	4	45,5	910
				9,1		3,9		
				9,9		2,9		
31	2,22	2,7	47	10	4,2	3,6	29,6	518
27	2,1	4	80	9,3	9,7	3,2	33,6	672
22	1,71	3,5	56	9,6	1,6	4,2	14,3	229
36				9,8		3,8		
				8,7		3,6		
26	1,94			9,8		3,9		
32				8,9		3,7		
27	2,1	2,2	44	8,9	0,7	3,4	22,4	448
28	2,06	2,8	48	10,1	3,7	4,4	26,3	447
41	1,68	3,5	66,5	9,6	4	3,3	33,5	637
29	1,85	1,5	36	9,6	0,5	3,5	22,6	542
33	1,8	1,3	32,5	8,9	0,7	3	32,7	818
63	2,19	4,3	120,4	9,6	9,2	2,9	44	1232
52				9,7		3,6		
	2,11			9,3	14,5	2,3		
44	2,05	7,6	114	9	9,1	3,8	68	1020
38	1,9	2,9	52	9,3	3,4	2,9	18,5	333
				9,5		4,1		
55	2,02	1,9	20,9	9,9	7	3,6	75,9	835
				9,6		3,6		
25	2,04	5,6	84	9,7	3,8	2,7	34,1	512
28	2,02	2,5	36,25	10,3	1,2	3,4	20	290
46	2,4	2,8	48	9,3	0,8	3,9	37,1	631
				9,5				
62	2,06	5,5	82,5	10	5,4	3,5	53,8	807
33	2,33	3,5	88	9,5	6	2,9	64,2	1605
46	2,35	2,2	29,7	9,6	2,9	3,7	59,9	809
62				10		3,3		
29	1,95	4,9	88	10,1	3,3	4,5	27,8	500
39	1,64	4,8	62	9,4	5,9	3,3	50,6	658
				9,5		3,2		
47	2,03	3,6	54	9,8	2,9	3,6	36,6	549
24	1,35	0,9	26,1	9,9	0,9	3,5	16,4	476
38	1,8	3,5	63	9,4	9,7	3,2	54,9	988
37	1,86	1,1	18	9,6	0,5	2,2	21,6	346
24	1,98	2,4	47	9,5	1,5	3,2	30,8	601
25	1,7	4,1	115	9,3	11,6	3,3	31,4	879
39	1,6	2,2	33	8,8	0,8	2,8	31,8	477
37	1,8	5,6	112	9,3	1,6	3,5	34,8	696
78	1,8	6,1	109,8	9,9	17,7	3,3	50	900
34	1,73	5,7	82,65	9,1	0,4	2,6	48,2	699
38	2,19	4,3	109,65	9,3	0,6	3,7	30,8	785
44	1,88			9,3		3,5		
31	2,22	3,2	54	9,2	2	3,7	43,5	740
24	1,86	2,1	37	9,9	2,5	3,3	20,8	364
25	2,26	5,9	133	9,2	2,3	3,4	28,1	632

32	1,61	4,7	94	9,9	19,7	3,3	28,7	574
38	2,11	6,9	100	9,3	4,7	4,2	46,6	676
53	1,8	5,6	95,2	9,6	6,7	3,1	29,6	503
36	2,1	5,4	99,9	9,7	4,7	3,3	39,6	733
				9,2		2,5		
30	1,74	4,2	80	9,3	2,7	3,4	38,2	726
40	1,72	3,6	70,2	9,5	17,6	3	43,8	854
40	1,78	3,1	43	9,3	7,7	3,8	38,5	539
59				9,8		3,6		
				9,6		4,4		
				9,1		3		
84	2,04	8,9	115,7	9,5	14,7	3,4	84,9	1104
	2,43			9,6		3,2		
	2,09			8,9				
				9,6				
				9,7				
41	1,79	4	64	10,1	17	3,2	59,8	957
43	2,09	3,5	50,4	9,3	3,6	4,1	32,1	465
38				9,7		3,5		
				9,3		3,2		
				9,4		2,6		
43	1,97	10,8	194,4	10,1	13,8	3,8	60,7	1093
36	2,06	1,4	42	10,4	8,3	4,1	25,3	759
44	2,25	2	34	9,8	2,5	2,8	41,3	702
	2,14	7,1	85	9,4	10,9	2,6	39,5	474
11	1,8	0,6	11	9	3,5	4	8,8	167
	1,82			9,6		1,9		
51	1,6	6,3	119,7	9,1	16,7	3,9	20,2	384
				9,3		3,4		
25	2,15	5,2	98,8	10,1	15,3	3,8	41,7	792
	1,9			9,1		4,1		
				9,2		2,9		
34	1,91	2,2	52,8	9,6	0,8	2,5	37,6	902
	1,77			9,6		2,6		
24	2,36	3,7	59	10,3	2,7	3,5	18,2	291
78	2,04	11,9	131	10,1	10,9	3,8	76,7	844
32	2,1	2	46	8,8	1,6	3,1	30,6	704
63	1,98	4,2	48,3	9,8	7,9	4,5	25,1	287
20	2,33	1,6	32	9,5	1,8	3,7	20,9	418
37	1,98	5	45	9,4	2,3	4,2	46,1	415
37	1,69	6,8	102	9,3	0,7	3,2	39,5	593
	2,15			9,6		4,3		
34	1,98	6,7	190,95	9,7	0,6	3,3	36,6	1043
48	2,16	2,7	66	9,4	1,8	3,4	39,4	965
67	1,8	5,7	65,55	9,1	9,3	3,5	42,9	493
	2,12			9,6		3,1		
68	1,93	1,9	18	9,5	4,2	3,1	45,3	430
74	1,48	5,1	66,3	9,4	1,3	4	17,4	226

29	1,99	7,9	107	9,2	6	3,1	23,7	320
41	2,02	5,5	102	8,7	5,7	4	38,7	716
35	2,39	3,7	92,5	9,8	3,9	3,7	41	1025
				9,4		2,8		
36	1,48	7,1	124,25	9,9	12,7	2,8	61,3	1073
40	2,08			8,9		2,8		
38	2,03	2,8	50	9,1	0,7	3,3	23,6	425
	1,89			8,9		2,9		
				9		2,4		
48	2,4	3,6	70	9,2	0,5	3,9	28,1	548
42	1,68	4,5	119	9,4	8,3	3,5	27,3	723
11	1,89	1	18	9,9	2,3	2,7	22	396
39	2,1	4,5	72	9,2	2	4,2	41,3	661
33	1,94	3,3	64	9,6	1,4	3,8	32,4	632
55	1,94	4,1	62	9,1	5,6	3,8	38,6	579
32	1,68	1,5	52,5	9,9	1,5	4,7	27	945
44	1,55	2,7	45,9	9,6	2,6	3,6	33,8	575
28	1,95	1,8	48,6	10	8,2	2,2	32,3	872
44	1,6	4,4	79,2	9,3	3,8	3	41,5	747
37	1,57	3,8	87,4	8,8	7,2	2,7	35,1	807
53	1,84	3,4	68	10,1	3,8	2,6	33,4	668
49	1,86	8,4	84	9,7	7,5	3,2	60	600
34	2,19	1,2	24	9,6	2,1	3,1	25,6	512
36	1,96	2,8	42	9,9	1,3	3,3	36	540
26				9,8		2,7		
				9,5		3		
	2,2			9,4		3,6		
37	1,8	2,6	72,8	8,9	2,6	3,4	39,3	1100
47	1,85	2,8	50,4	9,7	9,1	3,3	20,7	373
75	1,92	6,9	103,5	9,7	6,8	3,4	47,5	713
35	1,8			8,3		3,9		
				10,1		3,8		
	1,97			9,3		4,2		
34	2	4	42	9	4,1	3	38,6	405
	2,31			9,9		3,2		
34	1,88	1,8	41	9,5	0,6	3,3	17,8	402
21	2	2,9	49	10,3	3,5	3,7	36,9	627
23	1,79	1,5	30	9,4	7,3	2,5	30,7	614
48	1,91	4,5	65	10	2,9	3,3	39,4	571
41	2,14	6,8	102	9,4	3,3	3,9	56,8	852
28	2,01	3,1	58,9	9,9	2,1	2,2	27,3	519
45				9,7	6,5	3,6	31	403
60	1,8	11,5	115	8,9	7,3	3,3	61,1	611
41	1,9	3,3	63	9,4	0,7	3,5	27,2	517
52	1,91	2,9	45	9,6	2,3	3,8	27,7	429
34	1,9	7,1	71	9,9	9,1	4,3	22,1	221
27	2,17	4,9	98	9,3	2,8	3,2	31,3	626
27	2	3,5	95	9,2	10,8	2,2	18,5	500
21				9,1		3,1		
				9,5		2,7		
28	2,51	4,9	95,55	9,6	0,4	4,3	26,8	523
8				9,4		4		
33	1,92	2,6	44,2	10,4	4	3,4	22,1	376
				9,2		4,2		

55				10,4		3,7		
46				9,6		4,5		
34	1,9	2,3	41,4	9,6	0,4	3,2	20,1	362
34	1,8	3,5	54,25	9,3	6,4	3	18,9	293
60	1,82	5	60	9,5	1,6	4	70	841
37	1,8	3,1	62	9,2	3,2	3,4	33,5	670
13	2	2,5	80	8,5	1,1	2,6	24,1	771
49	2,06	3,6	136,8	10,4	6,4	3,4	35,3	1341
				10,2		3,5		
				9,2		4		
4	1,82	2,4	68	10,1	3,8	4,3	8,2	234
27	1,77	3,2	64	8,6	1,2	3,8	39,9	798
56	2,18	5	78	9,1	1,5	3	41,6	645
21	2,52	4,3	43	10	0,9	3,3	20,9	209
25	1,93	2,4	48	9,9	1,4	3,6	30,3	606
	2,12			9,6		3,4		
24	1,9	5,5	99	8,7	12,5	4,1	76,9	1384
66	1,96	11,6	98,6	9,7	17,7	4,4	76,5	650
27	1,84	7	196	9,9	2	4,8	26,4	739
29	1,4	2,5	50	10,1	5,2	3	36,3	726
30	2,16	6,2	81	9,2	4	3,3	46	598
17	1,78	4,8	79,2	9,7	6	4,1	24,3	401
31	1,67	4,3	101	9,6	8	3,2	45	1058
7	1,9	0,8	21,2	9,7	0,4	2,9	16,3	432
25	2	2,7	64,8	9,8	1,8	3,8	27,4	658
25	1,64	2,2	70,4	9,2	2,6	3,4	23,7	758
27	2,1	1,9	33,25	9,7	4,2	3,7	30,7	537
15	2,02	5,1	87	9,5	1,3	3,5	17,9	304
24	2,26	3,6	86	8,4	2	3,9	28	672
29	2,07			8,8		3,7		
28	1,9	5,2	104	9,3	2,5	2,8	37,3	746
19	1,21	1,9	57	9,6	0,6	4	33,1	993

Alkaline Phosphatase	TIBC	PTH	Vit D 25	Vit D 1.25	Vit B12	Folic Acid	TSH
52	248	154,3	28,27		373	6,58	1,659
82	282	571	10,43	15	490	12,15	1,8
84							4,42
78	295	45,8	27		243	>24	1,937
95							4,047
140	337	43	14,45		500	16,3	3,281
46	343	43,6	32,79	49			2,522
70	304	40,4	47,9				
72	312	47,6	29		345	13,24	0,64
	260			53	361	10,2	4,113
106	284	75,3	10,55		208	6,23	4,882
66	316	39,3	12,3				
86	302	54,4	14,94		314	4,26	2,18
68	253	13,6	15,48	27	711	8,7	1,42
113	353	75,5	19	31	213	11,23	3,954
111	248	200	30		646	5,65	3,493
119	314	89,2	8,49		408	7,31	1,827
80	304	49,8	17,1		370	8,51	3,73
105	277	74,6	14		257	18,82	
71	267	107,9	24				1,55
68	281	111,5	10,64		320	5,8	0,326
75	301	194,5	17	25	334	10,41	1,746
66	309			48	727	11,86	2,393
110	325	49,4	48				2,12
100	270				856	12,1	2,706
56	387	47,5	8,09		181	2,6	0,961
68	269	60,7	20		409	7,8	5
94	348	39,1	15,67		276	7,43	3,464
73	335			29	524	16,26	3,68
56	290	54,5	14,79		351	9,7	1,243
64	418	31,2	52	47	237	11,8	2,29
18	358	40,7	34		493	9,6	0,76
62	324	77	38		706	7,29	1,678
69	309	43,1	26	42	716	4,89	4,27
87	340	49,2	37,81	14	398	4,09	1,731
96	292						1,72
64	251	32,7	25	36			
69	264	81,5	26	23	254	8,1	
79	359	42,3	41	53	361	3,89	2,61
57	400	172,4	6,2	32			4,48
73	347	82,2	15,93	40	280	4,46	3,318
52	312	38,1	14,3	36	380	9,91	1,84
51	273	64,9	31,23		234	4,16	2,15
59	319	130,3	31	27	227	7,32	1,14
66	406	38,3	7,7	33	363	9,16	0,766
107	321	190,2	19		426	7,92	1,800
93	265	197	15,04		376	>24	4,467
57	330	68,1	19,11		429	5,83	1,331
55	283	94,1	32	49			
79	287	50,4	24		256	9,21	3,77
96	359	60	25	81	291	4,72	1,44

65	334	39	40	41	564	20	1,224
85	311	47	20,11				
69	381	40,5	31,33	45	373	11,2	4,315
	319				415	3,44	3,238
56	375	60,5	32,12		390	9,38	0,591
85	287			71	818	12,02	2,046
100	324	59	9,28		429	14,28	2,528
52	451	43,4	12	10	223	3,51	1,980
59	378				411	7,19	3,33
39	415	29,3	10,62		295	3,1	0,34
52	394	65,9	31		296	4,64	1,65
85	328				597	7,46	2,979
86					310	3,05	1,258
92	259	44,3	16,11		505	5,72	0,99
42	427	41,7	11,64	64	315	4,07	2,209
64	264		10,55		527		1,04
92	309				606	5,65	1,027
93	287				606	12,24	2,727
51	417	38,9	4,97	36	388	4,77	1,603
64	269	29,8	20,53	62	384	9,46	3,055
72	273	42,6	21		461	6,97	1,047
101							2,347
							0,85
103	301	51			343	7,89	1,451
55	333	19,7	20	36	348	5,77	1,22
67	588	14,6	23,48		411	8,28	1,350
103	340	51,6	19,4		473	13,56	1,495
41							1,172
120	352	78,8	74		716	12,42	1,95
116	250	117,4	13	29	384	7,43	0,988
60	326	32,8	15,77		465	10,25	2,221
74	389	25,3	33,34	30,9	405	5,87	0,776
62	350				381	4,07	3,915
73	451	10,9	14,09		562	7,33	2,64
148	201				592	12,7	2,067
49	279	61,8	22		568	8,07	2,824
71	218	65,9	17		760	6,06	8,914
92							3,157
94	448	13	20,74		508	8,66	4,639
59	351	50,5	25	30,71	354	12,8	3,31
91	406	40,4	12,36	28	640	22,82	0,78
121	359	87,4	17,18	44	411		3,35
60	292	23,4	10		458	8,27	1,771
107	236	73	25		667	13,4	3,01
115	281	102,7	7	43	404	4,73	6,22
100	269	103	15,42		462	4,74	3,412
47	263	104,1	28				
77	386	111,5	17,11	28	465	11,59	1,58
49	311	31,1	17,63		1031	8,52	1,91
58	359	48,7	22,29		299	11,48	1,908
75	312	155,4	23,8		555	6,84	3,878
47	273	27,8	28,6		101	10,15	4,81
71	319	69,7	3,95	28	244	2,79	3,25

82	287	114,1	16,16	49	486	22,07	1,438
64	290	91	9,88	26	314	15,68	5,069
143	431	125,5	16,03		636	8,74	1,42
45	298	76,8	14				3,12
77	328	25,9	12	23	370	7,52	2,39
100	386	80,2	30,74		525	5,98	0,859
82	304	57	27,32	47	292		2,344
80	295	37,6	31,49		419	7,62	7,614
94	249				323	4,88	1,617
57	278	134	20,86		468	7,33	
90	276	97,9	16,63	30	276	15,08	2,309
78	337	51,5	9		387	5,55	4,66
42	321	54,5	13,6	14	420	10,7	1,374
97	282	36,9	21		492	15,39	3,703
141	403	154,4	13,15		304	13,3	3,684
59	324	94	24		371	10,39	3,129
76	326	27	26		282	14,89	3,527
57	391	41	17,58	37	456	3,97	1,688
80	284	104,5	28,23		610	23,47	3,995
72	356	37,9	10,46		339	7,13	1,812
86	348	12,6	5		247	4,37	0,257
80	443	31,3	11,81	21	605	16,41	2,358
56	314	31,9	16,56	27	314	8,61	1,893
104	319	83	15,08		396	6,980	
34	334	22,8	25		399	5,21	1,23
119	354	53	16		189	6,74	0,659
50	331		18		519	5,75	0,96
63	271	46,9	12,77		229	12,77	1,974
94	348	40,3	22	36	347	4,61	1,35
98	283	39			766	16,42	1,118
60	236	93,4	9,69		306	4,84	5,022
68	352		16		423	4,12	2,336
166	272	166,6	18,97		399	>24	2,42
103	357	87	23,97				1,817
97	361	48,7	17		218		2,968
55	464	99,1	12,16		260	9,09	1,55
47	328	63	14,5		336	6,79	2,566
81	250	20,8	33		470	11,5	1,23
80	315	71	20	20			2,19
85	307	69,6	9,03	24	294	13,5	6,247
44	384	37,5	11,31	32	356	4,18	2,739
47	333	116,7	13		540	8,14	0,918
74	278	46	35,3	36	150	8,73	1,32
72	315	170,8	11,45	27	487	10,61	4,927
88	354	125,9	41	47	569	12,39	3,248
59	282	18,8			291	8,75	
87	339	31,2	17		398	7,5	1,91
68	401	47,2	23,18		300	18,5	
30	394	24,2	25		338	6,49	2,04
68	288						1,945
120	268	203	11	34	416	7,52	0,976
53	307	28,3	40		699	6,24	2,564
57	353	28,6	33	18	409	9,49	0,62
77	371						2,723

86	389	71,2	41		465	8,49	1,62
72	307	21	14	14	341	8,99	3,231
86	263	154	10	28	327	5,6	1,735
68	315	57,8	23,46		505	8,1	3,49
161	428	257,7	15	7	304	12,03	1,002
60	297	105	14,57		421	8,82	3,172
79	281	60	27	27	534	8,24	2,049
38	357	11,6	24		456	12,2	1,77
62	461	33,2	16		354	>24	1,128
90		54,5	30,51				2,980
68	611	44,1	11,13	35	378	4,53	2,132
110	272	62,2	13,09	22	4,22	4,34	4,055
74	312	149,8	26		289	6,66	3,659
109	246	45,3	4,12		165	5,35	3,35
82	325	58,5	6,66	38	575	9,34	1,75
53	312	33,1	22		305	11,52	1,674
149	288	15,02	17		359	9,8	4,504
58	329	41,9	10	21	353	4,65	1,06
95	282	33,1	43,57	54	297	5,53	1,733
65	446	20,1	13,65		482	9,13	1,39
47	334	118,4	14,39		599	16,7	1,077
71	258	44,5	34,85		425	4,19	0,76
48	378	21,5	18,35		393	4,58	1,78
68	381	98	11,85		351	3,58	3,68
60	267	35,2	15,57		230	4,55	0,432
67	267	163,4	17	57	394	6,38	3,34
119	391	26,5	24,89		657	23,99	1,898
108	335	84,1	4,91		364	6,96	4,260
73	236	67,7	7,68		291	6,37	2,540
109	315	64,8	16,26		379	6,51	3,409
64	267	31	9,26		889	6	2,49
111	301	79,2	16	33	499	5,43	3,8

T3	T4	Vit A	Vit E
3,53	1,16	1,28	12,2
	1,45		
3,13	1,07		
2,73	1,16	0,6	12,6
3,70	1,25		
3,28	1,44		
	1,03		
2,70	1,19	0,38	11,9
2,91	1,01	0,8	19
2,49	1,11	0,81	17,1
3,05	1,3	0,66	14,1
3,09	1,32	0,78	14
	1,25		
2,91	1,21	0,57	14,2
3,28	1,13	0,81	11,9
	1,04		
	1,29		
3,56	1,07		
3,81	1,33		
2,98	1,16	0,8	11,4
2,95	1,1		
3,45	1,22	0,44	13,6
2,81	1,05		
2,68	1,37	0,6	12,2
3,15	1,31		
2,51	1,12	0,75	15,5
3,24	1,47	0,86	10,3
	1,45		
2,90	1,03		
2,75	1,09	0,7	15,3
3,39	1,09	0,69	15,7
3,17	1,18	0,67	11,6
3,84	1,21	1,03	15,7
2,62	1,07	0,29	10,7
3,86	1,15	1,02	19,9
2,22	1,11	0,88	8,4
2,55	1,54	0,44	8,3
3,11	1,22	0,69	13,2

3,97	2,39	0,93	9,9
3,44	1,19	0,55	17,3
4,05	1,01		
2,95	1,22	0,51	12,5
2,97	1,27	0,36	13,5
1,36	1,17	0,38	8,2
3,22	0,95		
2,23	1,46	0,53	13,8
4,16	0,99		
3,06	1,18		
	1,15		
3,02	1,15	1,4	24,6
		0,65	11,3
2,97	1,08		
3,16	1,17		
2,91	1,00	0,92	24,9
3,28	1,15	0,52	12,6
2,75	1,33		
3,71	0,92	1,01	25,4
	1,5		
3,44	1,19	0,75	12,4
3,15	1,25		
3,04	1,08	0,89	16,6
2,83	1,44	0,9	21,7
3,76	1,42	1	12,7
	1,31		
4,12	1,48		
3,12	1,25	0,9	12,2
3,07	1,27		
3,62	1,26	0,55	13
2,92	1,69	0,5	16,6
3,05	1,21	1,03	17,2
3,09	1,17	0,46	9,2
	1,10	1,29	13,1
3,30	1,26	0,53	9,4
3,78	1,22	0,83	8,6
2,90	1,02	0,7	16
	1,73		
2,46	1,13	0,63	12,1
2,51	1,47	0,73	9,6

2,68	1,15		
2,59	1,59	0,5	14,7
2,78	1,49		
3,68	1,21		
	1,31		
3,04	1,15		
3,52	1,04	0,76	12,3
	0,82		
2,81	1,19		
3,34	1,30		
3,57	0,92	1,14	18,9
	1,07		
2,97	0,86	0,54	14,3
3,41	1,26		
4,19	1,39	0,78	14,7
	0,95		
3,29	1,17	0,78	13,5
2,59	1,07	0,66	12,2
2,81	1,07	0,35	11
2,77	1,73	0,86	10,8
3,31	1,1		
3,10	1,24	0,74	23,2
3,74	1,27		
3,12	1,11		
		0,76	7,6
3,07	0,94	0,94	17
		0,52	16
2,96	1,46		
2,68	0,99	0,54	11,58
3,08	1,04		
	1,19		
	1,11		
2,9	1,06	0,75	15,3
2,88	1,23		
3,56	1,26	0,61	11,2
3,05	0,93		
2,90	1,24		
2,76	1,13	0,5	17,2
3,53	1,42		
2,65	0,97	0,78	25,4
	1,23	0,95	19,2
2,98	1,29	0,82	11,3
2,89	1,23	0,97	13,1
3,16	1,27	0,63	10,2
3,14	1,17	0,45	15,7
	1,08		
1,97	1,10		
3,47	1,14	0,6	13,2

3,36	1,24		
3,52	1,08	1,01	15,5
3,33	1,39	0,51	16,2
3,04	1,04	0,62	3,04
4,01	1,19	0,44	16,6
2,83	0,86	1,56	12,3
1,23	1,05	0,66	12
3,45	1,06		
3,05	1,20	0,43	13,4
3,32	0,95	0,76	10,9
3,05	0,96	1,15	10,3
2,92	1,17	1,02	19,6
3,22	1,08	1,14	14
2,97	0,88		
3,76	1,17	0,56	19,4
3,1	1,27	0,58	14,6
4,30	1,53	0,73	11,8
3,44	1,23	0,84	23,6
3,14	0,97		
2,78	1,14	0,81	23,5
3,20	1,08	0,95	14,1
3,13	1,3	0,43	8,3
2,89	1,24	1,1	18,9
2,60	1,14		
3,55	1,79	0,6	8,5
2,54	0,93	0,68	13,5
2,81	1,11		
	0,88		
2,55	1,11	0,27	9,4
3,61	1,32	1	19,4
